# Cell–cell coupling and DNA methylation abnormal phenotypes in the after-hours mice

**DOI:** 10.1186/s13072-020-00373-5

**Published:** 2021-01-06

**Authors:** Federico Tinarelli, Elena Ivanova, Ilaria Colombi, Erica Barini, Edoardo Balzani, Celina Garcia Garcia, Laura Gasparini, Michela Chiappalone, Gavin Kelsey, Valter Tucci

**Affiliations:** 1 Genetics and Epigenetics of Behaviour (GEB) Laboratory, Istituto Italiano Di Tecnologia, via Morego, 30, 16163 Genova, Italy; 2 Epigenetics Programme, The Babraham Institute, Cambridge, UK; 3 Neurodevelopmental and Neurodegenerative Disease Laboratory, Istituto Italiano Di Tecnologia, via Morego, 30, 16163 Genova, Italy; 4 Neuroscience and Brain Technologies, Istituto Italiano Di Tecnologia, via Morego, 30, 16163 Genova, Italy; 5 Present Address: AbbVie Deutschland GmbH & Co, Knollstr, 67061 Ludwigshafen, Germany; 6 Present Address: Rehab Technologies, Istituto Italiano Di Tecnologia, via Morego, 30, 16163 Genova, Italy; 7 Present Address: Brain Development and Disease, NBT, Istituto Italiano Di Tecnologia, via Morego, 30, 16163 Genova, Italy; 8 Present Address: BioMed X Innovation Center, Im Neuenheimer Feld 515, 69120 Heidelberg, Germany; 9 Present Address: Center for Neural Science, New York University, New York, NY 10006 USA

## Abstract

**Background:**

DNA methylation has emerged as an important epigenetic regulator of brain processes, including circadian rhythms. However, how DNA methylation intervenes between environmental signals, such as light entrainment, and the transcriptional and translational molecular mechanisms of the cellular clock is currently unknown. Here, we studied the after-hours mice, which have a point mutation in the *Fbxl3* gene and a lengthened circadian period.

**Methods:**

In this study, we used a combination of in vivo, ex vivo and in vitro approaches. We measured retinal responses in *Afh* animals and we have run reduced representation bisulphite sequencing (RRBS), pyrosequencing and gene expression analysis in a variety of brain tissues ex vivo. In vitro, we used primary neuronal cultures combined to micro electrode array (MEA) technology and gene expression.

**Results:**

We observed functional impairments in mutant neuronal networks, and a reduction in the retinal responses to light-dependent stimuli. We detected abnormalities in the expression of photoreceptive melanopsin (OPN4). Furthermore, we identified alterations in the DNA methylation pathways throughout the retinohypothalamic tract terminals and links between the transcription factor Rev-Erbα and Fbxl3.

**Conclusions:**

The results of this study, primarily represent a contribution towards an understanding of electrophysiological and molecular phenotypic responses to external stimuli in the *Afh* model. Moreover, as DNA methylation has recently emerged as a new regulator of neuronal networks with important consequences for circadian behaviour, we discuss the impact of the *Afh* mutation on the epigenetic landscape of circadian biology.

## Introduction

Gavin Kelsey and Valter Tucci jointly directed this work.

The regulation of the 24-h circadian rhythms of many physiological processes, such as sleep–wake cycles, behavioural responses, and endocrine modifications, depends on the entrainment of the internal clock with environmental signals, the most important of which is light [1]. In complex multicellular organisms, the internal clock is implemented by interlocked transcriptional and translational molecular mechanisms and is cell autonomous. Furthermore, it presents a hierarchic organization throughout each organism [2]. Notably, the suprachiasmatic nuclei (SCNs) are the master clocks of the brain and function as pacemakers for other brain areas and for peripheral organs [3].

In mammals, such as humans and mice, non-visual light signals detected by the retina activate molecular events along the retinohypothalamic tract (RHT) that convey the external environmental information to the SCN. The transfer of information between the environment and the central internal clock is accomplished by the photopigment melanopsin, encoded by the gene *Opn4*, at the intrinsically photosensitive retinal ganglion cells (ipRGCs) of the retina. The activation of this molecular system leads the main neuronal response to light-entraining processes [4]. However, environmental light conditions exhibit both short- and long-term variations and, incidentally, the non-image-forming visual processing system interact with the classical visual cellular system to regulate dynamic intrinsic mechanisms that eventually maintain entrainment with the light–dark (LD) cycle [5]. The mechanisms that participate in this process and their key components and interactions remain still partially unknown and can be investigated in either side of the visual system.

Mouse models have been used to study circadian biology for years. Investigations of light processing pathways and their contributions to circadian core mechanisms, remain a valuable experimental model to study new avenue in circadian biology, such as the epigenetic landscape of the biological clock. For example, DNA methylation has been proposed as a new mechanism that can adaptively modulate long-lasting phenotypic changes in the circadian clock [6–9]. It has been demonstrated that DNA methylation in the mouse SCN regulates behavioural responses to environmental variations (e.g., shortening or lengthening) of 24-h environmental light cycles [6]. In the absence of entrainment signals, this phenomenon is known to set the endogenous free-running clocks of mice according to the previous periodicity (producing ‘aftereffects’) [10]. Furthermore, it has been reported that DNA methylation is important in the regulation of neuronal networks [11]. Inhibition of the reprogramming of DNA methylation abolishes local dynamics between SCN neuronal networks [7], a process that is not region-specific but instead relies on the communication between neuronal networks. This interregional circuit is mediated by GABAergic signalling that supports the plasticity of the cellular clock and maintains the entrainment to LD cycles [7]. Therefore, a similar regulatory process is potential expandable outside the SCN, into other neuronal networks and local clocks.

Here, we studied the after-hours (*Afh*) mouse line: *Afh* mutant mice carry an A > T transversion in the *Fbxl3* gene that results in a Cys^358^Ser substitution within the F-box protein. The mutation delays cryptochrome 1 and 2 (CRY1-2) degradation and increases the stability of these proteins [12]. A clear role of Fbxl3 has been shown in ubiquitination mechanisms within the core molecular clock system related to lengthening of the free-running circadian period [12, 13]; deficits in sleep regulation and timed behaviours [14]; and impairment of cognitive and emotional behaviours [14, 15]. Moreover, the *Fbxl3* mutation is responsible for abnormal changes in neuronal excitability between light and dark in SCN neurons, leading to specific GABA-mediated hyperpolarization in the *Afh* ventral SCN (vSCN) [16].

In one of our recent studies, we showed that *Afh* mice can easily entrain with a longer LD cycle, as supported by a delayed onset of activity [14]. We demonstrated that mutants are unable to both adapt to progressive anticipation of the light phase and reduction of the dark phase, and to adequately perform cortical cognitive process. These results suggest that the oscillatory systems that regulate this circadian plasticity are disrupted between dark–light transition and that *Afh*-mediate mechanisms potentially impact on brain networks that control executive functions, for example cortical neurons.

To narrow down the study of the above phenotypes, we explored phenotypic responses at electrophysiological and molecular level. Therefore, we studied properties of cell–cell communication in vitro, in vivo electrophysiology and then focused on epigenetic and transcriptional processes.

## Results

### The Afh mutation impairs neuronal functional coupling

**Fig. 1:**
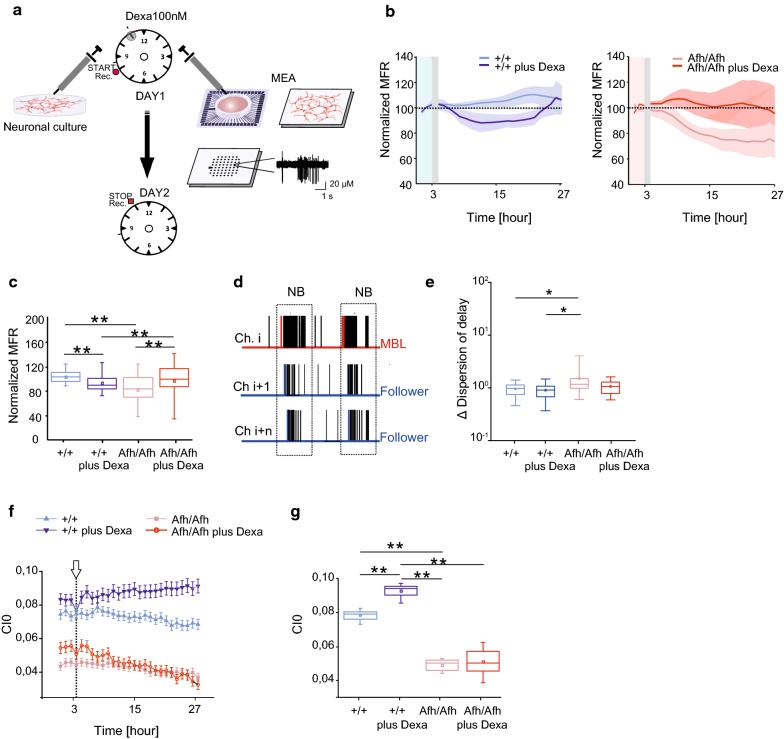
Afh/Afh primary neuronal networks show electrophysiological abnormalities. **a** Scheme of the in vitro treatment protocol carried out for gene expression and electrophysiological analyses using chronic treatment with 100 nM Dexa at different time points. The right panels show a 60-channel microelectrode array with a typical recorded trace. **b** (Left) Mean firing rate (MFR) of +/+ cultures recorded without stimulation (light blue line, *n* = 5; DIV = 22 ± 3) and after stimulation (blue line, *n* = 5; DIV = 25 ± 3). (Right) MFR of *Afh*/*Afh* cultures without stimulation (light red line, *n* = 5; DIV = 21 ± 3) and after stimulation (red line, *n* = 5; DIV = 22 ± 3). **c** Box plot of MFR for +/+ neuronal networks without treatment (light blue) and with treatment (blue) and for *Afh/Afh* neuronal networks without treatment (light red) and with treatment (red). The bold lines and shaded regions correspond to the mean ± SEM. **d** Spike trains from three different channels: the dashed windows indicate network bursts (NBs). For each NB, the channel exhibiting the first detected event was named the major burst leader (red trace), while the others (blue) were the followers. **e** Box plot indicating the normalized variation of the dispersion of delays calculated as the difference between the delay of the first and the last followers for +/+ neuronal networks without treatment (light blue) and with treatment (blue) and for *Afh/Afh* neuronal networks without treatment (light red) and with treatment (red). **f** Coincidence index (CI0, ± 3 ms) for +/+ neuronal networks without treatment (light blue) and with treatment (blue) and for *Afh/Afh* neuronal networks without treatment (light red) and with treatment (red). White arrow indicates moments of Dexa administration. **g** Box plot of CI0 for the +/+ and *Afh/Afh* neuronal networks without treatment (light blue line and light red line, respectively) and with treatment (blue line and red line, respectively). For each box plot, the small square indicates the mean, the central line illustrates the median, and the box limits indicate the 25th and 75th percentiles. The whiskers represent the 5th and 95th percentiles: * p < 0.05, ** p < 0.01. Statistical analysis was performed using two-way ANOVA with Tukey post-test

The molecular clock is present in cells, and cell functional coupling is an important factor dictating cycle length in large cell assemblies [17]. We investigated whether *Fbxl3* mutation affects neuronal network communication basic processes. To mimic the synchronized firing activity of SCN neuronal networks in vivo, we administered Dexamethasone (Dexa) to primary cortical neurons in vitro and we observed the effects on in vitro electrophysiological properties of primary neurons [18]. Dexa was administered 3 h after the beginning of the recordings. We used a chronic stimulation to mimic the effect in the brain. Clock genes responded to Dexa stimulation in wt neuronal cultures by showing a rhythmic, periodic expression which mimic circadian oscillations in vivo (Additional file 1: Figure S1A and Additional file 2: Table S1). We monitored the electrophysiological activity of in vitro neuronal networks for 27 consecutive hours using microelectrode arrays (MEAs; Fig. 1a). Dexa-induced stimulation immediately decreased the mean firing rate (MFR) in +/+ networks, which was recovered within 24 h (Fig. 1b, c, blue trace). Under control conditions, the *Afh*/*Afh* neuronal networks showed significantly lower MFRs than the +/+ networks (Fig. 1b, light red trace), suggesting that *Afh* neurons are less active than wild-type neurons. Moreover, in *Afh/Afh* neurons, external stimulation increased the firing rate of the network to a level similar to that of +/+ neurons (Fig. 1b, red trace) under control conditions.

Within a neuronal network, the firing of a specific set of neurons (major burst leaders, MBLs) leads the subsequent firing of additional neurons, generating a network burst sequence (Fig. 1d) [19, 20]. Investigation of these sequences is informative with regard to the dynamics of neuronal activity. Therefore, we analyzed the MBLs in *Afh/Afh* and +*/*+ cultures by assessing the temporal delay between the first active channel and the followers (Fig. 1d, e) during a network burst event. Analysis of the dispersion of delays showed that Dexa did not affect the time of signal propagation in +*/*+ networks; in contrast, compared with +*/*+ neurons, *Afh/Afh* neurons presented significantly higher dispersion under basal conditions that was reduced following Dexa treatment (Fig. 1e). The coincidence index (CI0), a statistical measure computed from the cross-correlation functions of any pair of neurons within a network, indicates the level of coupling (*i.e.*, temporal synchronization of activity) of the neurons within the network. In particular, we found remarkably low levels of cell–cell interaction in *Afh/Afh* networks compared to +/+ control networks (Fig. 1f, g). Furthermore, mutant neurons did not respond to external entrainment stimulation, indicating a reduced neuronal functional coupling profile due to the *Afh* mutation (Fig. 1f, g).

Thus, we adopted an up-to-date exhaustive stochastic mathematical model [21] developed to mimic the core cellular clock (Additional file 3: Figure S2A). Within the model, we varied the degradation of CRY until the output of the model mimicked the *Per* expression that we have previously reported in *Afh* mice [12]. Then, we used this index (Additional file 3: Figure Ssembles of *Afh* neurons versus wild-type control neurons. PER2 levels in *Afh* and +/+ were modelled and analyzed in silico neuronal networks over several 24-h cycles (Additional file 3: Figure S2C). The derived periods from all neurons showed a broader distribution in *Afh* neurons than in wild-type neurons (Additional file 3: Figure S2D), suggesting a higher variability of the circadian phenotype in mutants than in controls. After several simulations, the normalized levels of *Per* were dramatically reduced in *Afh *in silico neuronal analysis (Additional file 3: Figure S2E). Remarkably, the outcome of the simulations mirrored the *Per* expression profile that we observed in vitro while testing the *Afh*/*Afh* versus +/+ control cultures following Dexa stimulation (Additional file 3: Figure S2F).

These results highlight alterations in functional coupling properties of *Afh* neuronal networks that are only partially attenuated by external synchronization induced by Dexa. Strikingly, the mathematical simulations recapitulate the major molecular profile of the in vitro phenotype shown by the two different genotypes.

### Abnormal electrophysiological and molecular phenotypes are present in the retina and the SCNs of Afh mutants

Here we studied how *Afh/Afh* animals process light, with a particular focus during the transition between light–dark and vice versa.

**Fig. 2:**
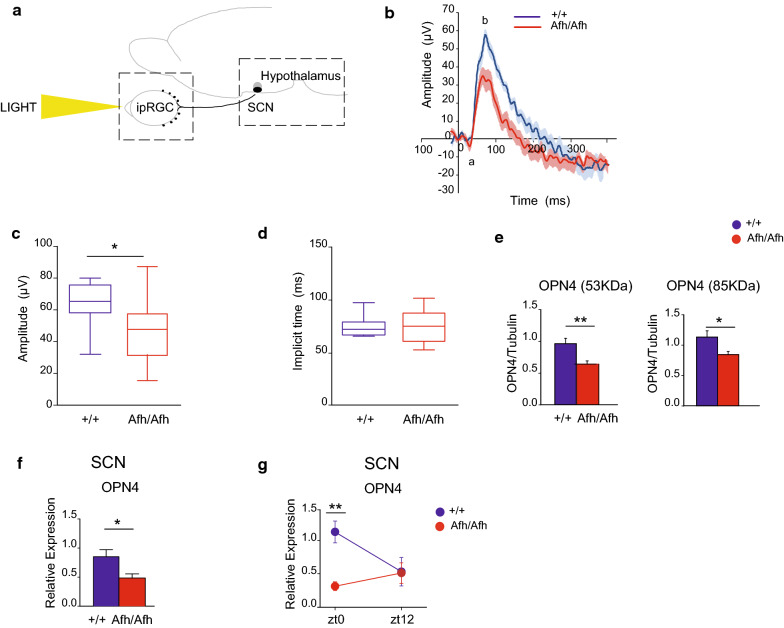
Altered cone response coupled with a decrease in OPN4 expression in the retina and the SCN of Afh/Afh mice. **a** Schematic representation of the RHT nodes, starting from the intrinsically photosensitive retinal ganglion cells (ipRGCs) to SCN and hypothalamus. **b** Flash electroretinogram (fERG) waveform of the cone response in *Afh*/*Afh* and +/+ animals (*n* = 14 for each genotype) measured in photopic conditions. The saturated negative response and the positive response are represented with letters a and b. **c** Box plot showing the quantified amplitude values of the b wave between the two genotypes. **d** Box plot showing the quantified implicit response times after fERG stimulation. **e** Quantification of western blotting for unglycosylated (53 kDa) and glycosylated (85 kDa) OPN4 isoform protein levels in the two genotypes (+/+ *n* = 13, *Afh/Afh* *n* = 11). OPN4 levels were normalized to β-tubulin levels. **f** *Opn4* expression levels in the SCN in *Afh* mutants (red) and controls (blue) (*n* = 25 +/+ , *n* = 24 *Afh/Afh*). **g** Relative expression of OPN4 normalized to multiple housekeeping genes at the two different time points, ZT0 and ZT12. The bars represent the average values for three different experiments ± SEMs (minimum *n* = 7 for each genotype at each time point). For each box plot (panels C and D), the small square indicates the mean, the central line illustrates the median, and the box limits indicate the 25th and 75th percentiles. The whiskers represent the 5th and 95th percentiles. * p < 0.05, ** p < 0.01, Student’s *t* test (**c**–**f**), two-way ANOVA plus Bonferroni post-test (**g**). All image analyses were performed with ImageJ software

Therefore, we decided to move retrogradely along the nodes of the RHT (Fig. 2a). Initially, we tested the retinal cone activity of *Afh* mutants and littermate controls by measuring the flash electroretinogram (fERG) response. The fERG is an electrophysiological measure predictive of the ability of the retina to respond to photoentrainment stimuli [22]. *Afh/Afh* and +/+ mice were exposed to uniform flashes of light of increased luminance generated by a Ganzfeld cupola, and the photoreceptor response was recorded using a gold electrode. A typical fERG waveform consists of ‘a’ and ‘b’ waves, which represent the activity of photoreceptors and bipolar cells, respectively. To measure the cone activity, we removed the rod contribution (i.e., the ‘a wave’) by saturating the background, as previously reported [22]. We exposed mice to the maximum stimulus of 30 cd s/m^2^ of luminance. We observed that the amplitude of the retinal response in *Afh* mutant mice was significantly decreased (*p* = 0.0124) as compared to that in wild-type mice (Fig. 2b, c), indicating that *Afh* mutants have reduced responses to light-dependent circadian inputs. The latency of the response was not affected in *Afh* mutant animals (Fig. 2d).

Considering that the main functions of the cone cells is associated with visual acuity, and non-visual photic entrainment is only a redundant function of these photoreceptors, it could still be possible that the differences we observed are only incidental to circadian entrainment.

As the photopigment Melanopsin is highly expressed in ipRGCs and plays a crucial role in maintaining circadian entrainment with the external environment by conveying non-visual light signals from the retina to the SCN [23, 24] we investigated OPN4 mRNA as well as the total protein levels in the retinas of *Afh/Afh* and +/+ mice at two different time points in a normal LD cycle, 08.00 (zeitgeber time, ZT0) and 20.00 (ZT12). Since mRNA levels isolated from an heterogenous source as the retina showed high variability (Additional file 4: Figure S3A), we focused our study mainly on protein levels. We first used an antibody able to detect both the unglycosylated (53-kDa) and glycosylated (85-kDa) forms of OPN4 in both wild-type and *Afh* mice (Additional file 5: Figure S4A). N-glycosylation can be involved in melanopsin maturation and localization. *Afh/Afh* animals showed significantly lower levels of both 53 kDa (*p* = 0.0046) and 85KDa (*p* = 0.0241) OPN4 expression compared to +/+ controls (Fig. 2e). Furthermore, levels of unglycosylated protein were significantly reduced by 30% in *Afh/Afh* animals compared with +/+ controls at ZT0, the beginning of the light phase (+/+ 1.000 ± 0.09; Afh/Afh 0.64 ± 0.098; *p* = 0.019), but not at ZT12, the end of the light phase (Additional file 5: Figure S4B). Instead, we observed significant reductions in the levels of the OPN4 glycosylated isoform in *Afh/Afh* mice at ZT12 (+/+ 1.032 ± 0.121; Afh/Afh 0.641 ± 0.132; *p* = 0.040) but not at ZT0 (+/+ 1.000 ± 0.112; *Afh/Afh* 0.812 ± 0.112; *p* = 0.248) (Additional file 5: Figure S4C). We then investigated on the presence of *Melanopsin* mRNA in the other terminal of the RHT tract, the SCN, due to the paucity of starting material for a biochemical analysis. The SCN tissue was identified using a *Six6*-specific transcriptional profile (Additional file 6: Figure S5A, B). *Opn4* transcript levels were lower in the SCNs of *Afh/Afh* mice than in those of +/+ mice (Fig. 2f: +/+ 0.85 ± 0.11, *Afh/Afh* 0.48 ± 0.07, *p* = 0.01). Interestingly, *Opn4* levels were low in *Afh* mutants at ZT0 but returned to wild-type levels by ZT12 (Fig. 2g). Immunohistochemical investigation of SCN slices revealed that OPN4 was expressed in both SCN cells and fibres (Additional file 5: Figure S4D).

Our data suggest an impairment in the expression and modification of both the glycosylated and unglycosylated forms of OPN4 at the transition between the light and dark phase and vice versa. *Afh/Afh* showed alterations of the fERG activity, in parallel we showed that OPN4 levels were altered also in the SCN of mutant animals at the same shifting moments between light and dark conditions.

### Epigenetic phenotypes: Fbxl3 mutation mediates specific DNA methylation changes, including of the Opn4 promoter, in Afh mutant SCNs

**Fig. 3:**
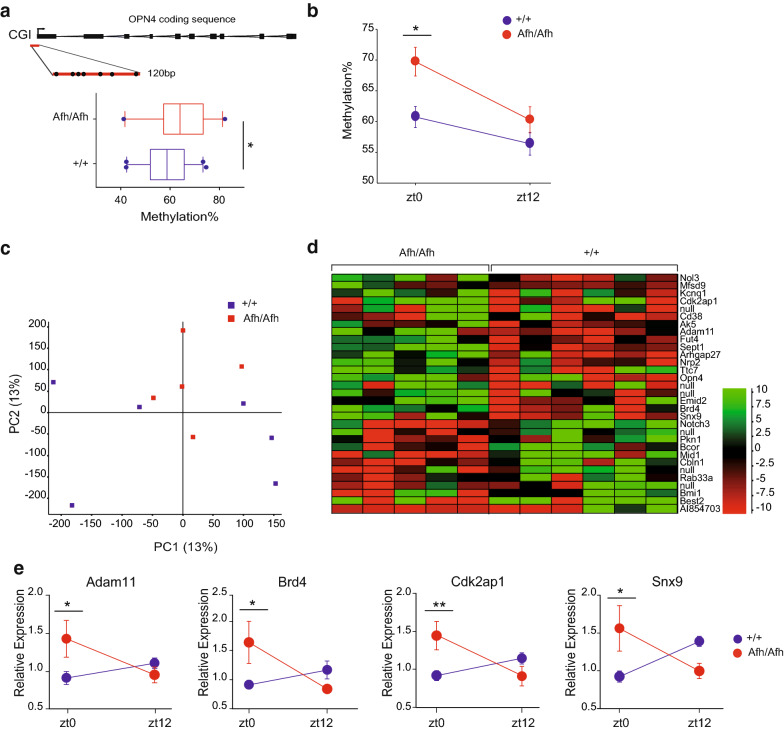
Discrete and specific differences in the methylation profiles of *Afh*/*Afh* SCN vs +/+ animals. **a** Schematic representation of the *Opn4* locus highlighting the CGI region (upper panel) at the beginning of the transcription start site (TSS, black arrow), as analyzed by pyrosequencing. Box plot of methylation values for each biological replicate averaged across all of the CpGs in the pyrosequencing amplicon (lower panel); the red lines represent the median, and the whiskers represent the 5th and 95th percentiles. **b** Pyrosequencing of *Opn4* CGI methylation levels in *Afh/Afh* and +/+ mice at the two different time points, ZT0 and ZT12. **c** Principal component analysis of CGIs methylation vs time of day for +/+ and *Afh/Afh* animals after RRBS sequencing. **d** Logistic regression of the RRBS data identified 31 differentially methylated CpG islands (CGIs) (with a difference greater than 10%). Quantitation of methylation levels was performed with the SeqMonk software suite considering CGIs with a minimum count of 5 per position and at least 3 observed positions for each CGI across the dataset. The coloured scale on the right indicates the hypermethylated CGIs (in green) and the hypomethylated CGIs (in red). **e** Relative expression of a subset of genes with differentially methylated CGIs identified by the RRBS analysis at the two different time points, ZT0 and ZT12, normalized to the levels of multiple housekeeping genes. The bars represent the average values of three different experiments ± SEM. * p < 0.05, ** p < 0.01, two-way ANOVA plus Bonferroni post-test (**b**), Student’s *t* test (**a**), two-way ANOVA with Tukey’s post hoc test (**e**). **a**–**b** *n* = 5 for *Afh/Afh* and *n* = 6 for +/+ . **c** *n* = 6 for each genotype. **d** *n* = 3 for *Afh/Afh* and *n* = 3 for +/+ at each time point. **e** Minimum *n* = 7 per genotype at each time point

Alterations in the OPN4 transcriptional levels in the SCN prompted us to investigate in further details the methylation status of this gene in the SCN. We performed quantitative bisulphite pyrosequencing for *Opn4*, in *Afh/Afh* and +/+ SCNs collected at both ZT0 and ZT12. An OPN4 CGI was localized in the promoter, a region of a gene body where methylation is associated with repression of transcription [25, 26]. Initially, we showed that a specific region of the *Opn4* promoter region was hypermethylated in mutants (Fig. 3a). *Afh/Afh* mice showed hypermethylation compared to wild-type mice (+/+ 58.57 ± 1.322; *Afh/Afh* 64.32 ± 1.322; *p* = 0.0105). The CGI cytosine residues were differentially methylated across the region, especially close to the transcription start sites (TSS) (Fig. 3a). Moreover, methylation level was dynamically altered between ZT0 and ZT12 in the two genotypes, with a significantly higher level at ZT0 in mutant animals compared to controls (*p* = 0.0243).

In order to have a secondary analysis, we conducted a complete genome wide investigation of methylation in the SCN. We profiled genome-wide DNA methylation in the SCNs of *Afh* mutants as compared to those of littermate controls through reduced-representation bisulphite sequencing (RRBS). RRBS enriches for CG-rich sequences, such as CpG islands (CGIs) that are associated with the majority of gene promoters and provides highly quantitative data with nucleotide resolution [27]. An average of 767,898 CpG sites with at least 5 reads were detected in each dataset (Additional file 7: Figure S6A and Additional file 8: Tables S3 and Additional file 9: Table S4). Of these CpG sites, an average of 66.3% were located inside CGIs, while 33.65% were located elsewhere in the genome (Additional file 7: Figure S6B and Additional file 8: Table S3). An average of 16,715 CGIs was covered across all datasets, with at least 15,562 CGIs detected in each sample (Additional file 8: Table S3). This coverage represents 67.6% of the CGIs reported for the mouse genome [28]. We used principal component analysis (PCA) to verify the clustering of the different datasets, which resulted in no obvious separation between the two groups according to genotype, (Fig. 3c). Next, we analyzed the data by combining all individual datasets by genotype (+/+ *n* = 6 and *Afh/Afh* *n* = 5), and used logistic regression to highlight differentially methylated CGIs. We looked then for methylation differences between the two genotypes greater than 10%. 3 out of the 31 differentially methylated CGIs identified in *Afh/Afh* (*Sept1*, *Notch3* and *Nrp2)* that matched known gene regions (Fig. 3d and Additional file 9: Table S4)*,* survived additional Replicate test for statistical difference.

The role of DNA methylation in gene expression control depends on genomic position. Approximately 50% of CGIs in the mouse genome are associated with TSSs, with the remaining 50% equally distributed between intragenic and intergenic areas [29], although these CGIs can demarcate alternative TSSs. We found that differences in DNA methylation occurred predominantly at intragenic CGIs (e.g., *Ttc7, Snx9, Cdk2ap1*, *Best2, Adam11*, *Opn4, Brd4* and *Sept1*), although some occurred at CGIs located in intergenic regions (Additional file 10: Table S5). We then further assessed the mRNA levels of genes identified by RRBS that are involved in cell–cell communication, rhythmic processes and transcriptional regulation, and we observed genotype-dependent increases in transcript levels at ZT0 for all the targets investigated, suggesting light-dependent activation differences of these genes between *Afh/Afh* and +/+ mice (Fig. 3e).

Our results indicate that differential methylation of a specific region of OPN4 promoter in *Afh/Afh* animals, is not associated with massive genome methylation differences as revealed by RRBS. Nevertheless, ZT0 increased expression of genes identified by RRBS and involved in cell–cell communication and coordination mechanisms, suggests an impairment of these pathways in the SCN, specifically during the dark–light transition.

### The expression of Dnmts and Tets is altered across the Afh peripheral terminals of the RHT

Because of the abnormal molecular, electrophysiological and epigenetic dynamics in *Afh* mutants, we sought to explore regulatory epigenetic processes across the RHT nodes. We focused on the expression of enzymes involved in DNA methylation and demethylation. DNA methylation is determined by different families of proteins [30]; DNA methyltransferase 1 (DNMT1) maintains DNA methylation, DNMT3s are responsible for de novo methylation [31], and the ten-eleven translocation (TET) proteins catalyze oxidative demethylation reactions.

**Fig. 4:**
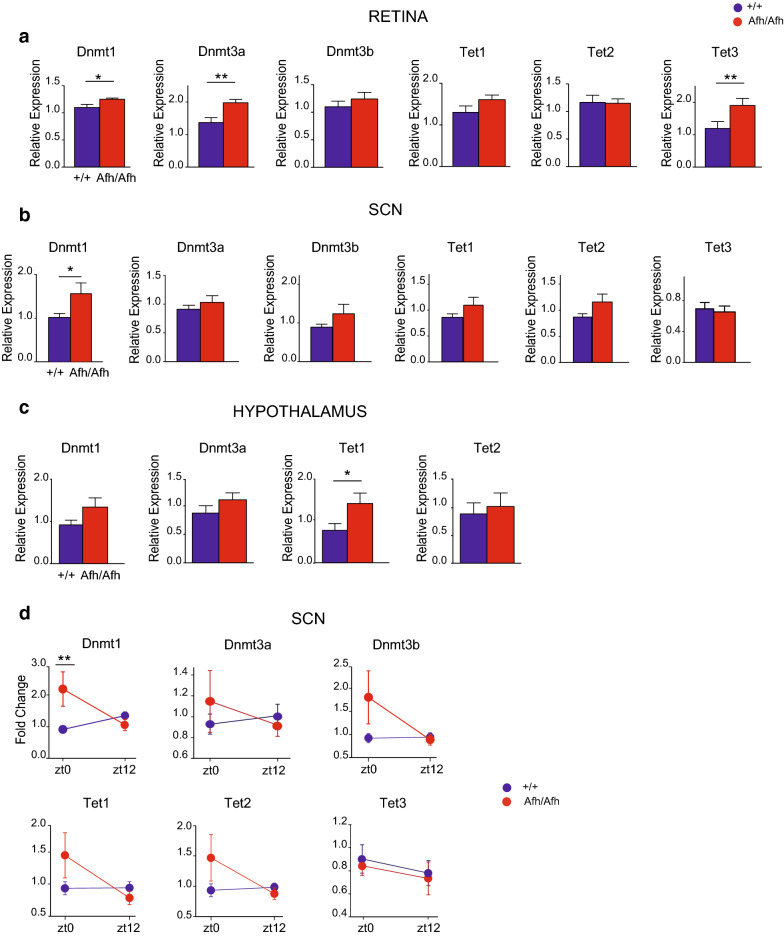
*Afh*/*Afh* mice show specific methylation enzyme expression anomalies in the different nodes of the retinohypothalamic tract (RHT). **a** Retinal expression levels of the transcripts for genes involved in DNA methylation in the *Afh/Afh* (red) and +/+ (blue) genotypes; (minimum *n* = 7 for each genotype). **b** Expression levels of methylation enzymes in the SCNs of *Afh* mutants (red) and controls (blue) (minimum *n* = 18 for each genotype). **c** Hypothalamic expression levels of the transcripts of methylation enzymes in *Afh* mutants (red) and controls (blue) (*n* = 12 for each genotype). **d** RT-qPCR of methylation-mediating enzymes at the two different time points, ZT0 and ZT12; (minimum *n* = 7 per genotype at each time point). All expression levels are normalized to those of multiple housekeeping genes. All bars represent the average ± SEM for at least two different experiments. * p < 0.05, **p < 0.01, two-way ANOVA plus Bonferroni post-test (panel D), Student’s *t* test (panels A–C)

We observed that *Dnmt1* expression levels were significantly higher in *Afh* homozygous mice than in wild-type mice in both the retina (Fig. 4a) and the SCN (Fig. 4b). An Increase in *Dnmt1* could be detected in the rest of the hypothalamus, although it did not reach significative difference (*p* = 0.28, Fig. 4c). *Dnmt3a* and *Tet3* mRNA levels were upregulated in the retina (Fig. 4a). In the hypothalamus instead only *Tet1* (Fig. 4c). These data indicated a specific dysregulation of methylation-mediated processes at the input and output of the circadian pacemaker (SCN and retina), but not to the rest of the hypothalamus.

To test whether methylation-mediating enzymes follow an expression pattern related to the LD cycle in the SCN, Retina and Hypothalamus, we compared time points at ZT0 and ZT12. We observed that the increased *Dnmt1* expression in *Afh/Afh* SCNs occurred mainly at the onset of light (+/+ 0.92 ± 0.38; *Afh/Afh* 2.18 ± 1.35; *p* = 0.0077), but not at the offset of light (Fig. 4d).

No difference in methylation enzymes between ZT0 and ZT12 was detected in the Hypothalamus (Additional file 4: Figure S3B). We observed instead similar result for *Dnmt1* and *Dnmt3a* in the Retina, both presenting an increased expression in *Afh/Afh* animals at ZT0 (Additional file 4: Figure S3C).

Our findings indicate that enzymes involved in de novo methylation (*Dnmt3a*), maintenance (*Dnmt1*) and active demethylation processes (*Tet1 and Tet3*) are altered throughout the RHT terminals of *Afh/Afh* mice, suggesting a change in the overall epigenetic landscape of these animals in concomitance with dark–light transition.

### Rev-ERBα controls the expression of DNA methylation-mediating enzymes

Finally, we sought to investigate how Fbxl3 is entwined with other proteins. At first, we examined the promoter regions (from 2 kb upstream to 500 bp downstream of the TSS) using Genomatix and the LASAGNA software suite to verify the presence of transcription factor binding sites (TFBSs) for members of the RORɑ family. Surprisingly, we found that most of the differentially methylated CGIs identified by RRBS, as well as promoters for genes encoding the methylation enzymes, particularly *Dnmt1*, *Dnmt3a*, *Dnmt3b* and *Tet1* and *Tet3,* presented multiple binding sites for RORα and REV-ERBα transcription factors (Additional file 11: Table S7). The score assigned to the core recognition sequence indicated the core sequence similarity (Additional file 11: Table S7) in the target region, while the matrix similarity score showed the degree of similarity between the target sequence and the Genomatix matrix. Our scores oscillated between 0.7 and 0.9, showing a good probability that the target sequences contained TFBSs for RORα and REV-ERBα transcription factors. Interestingly, it has been recently reported that Fbxl3 binds to Rev-ERBɑ by regulating its activity, in particular by attenuating its inhibitory effect on Rev response element (RRE) sequences [32].

**Fig. 5:**
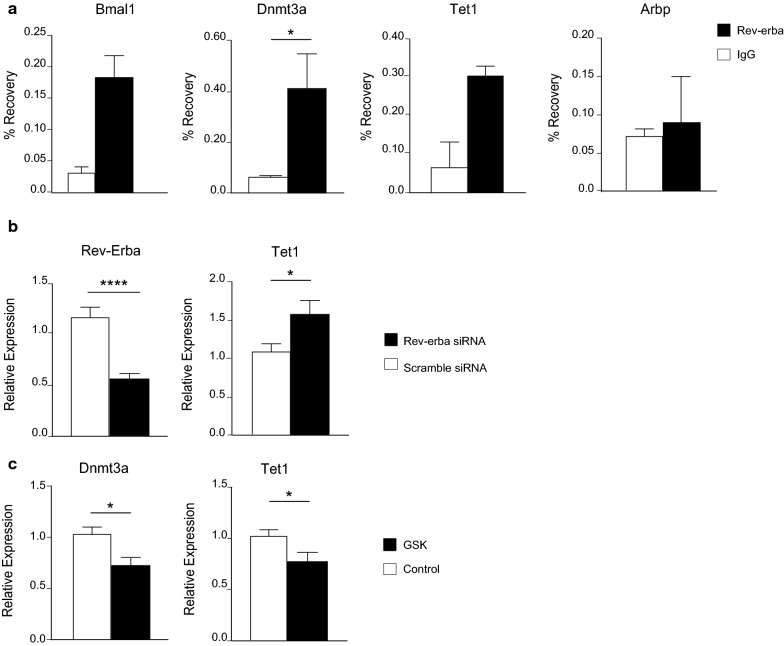
Rev-ERBα is involved in the transcriptional regulation of DNA methylation enzymes in vitro. **a** ChIP for Rev-ERBα in primary neuronal cells. Expression is normalized to the input. The bars represent the average values ± SEMs for two different experiments (*n* = 3 samples for each experiment pooled together for immunoprecipitation). **b** RT-qPCR of DNA methylation enzymes in primary neuronal cultures treated with a scramble siRNA and a specific siRNA against REV-ERBα. The bars represent the average values ± SEMs for three different experiments. **c** Drug treatment with the REV-ERBα agonist GSK 4112 at 10 μM of the drug on primary neurons of 8 DIVs (days in vitro). The bars represent the average values ± SEMs for two different experiments. The data were normalized on vehicle control (DMSO). * p < 0.05, ** p < 0.01, **** p < 0.0001, Student’s *t* test

Therefore, to better understand the link between Fbxl3 and Rev-ERBɑ, we focused our attention on wild type neuronal culture as the *Afh* mutation may bring with it heterogenous modulatory effects, and therefore introducing confounding factors into the system that we could not account for. We performed chromatin immunoprecipitation (ChIP) assays on wild-type primary neuronal cultures using a specific anti-Rev-ERBα antibody. *Bmal1* was used as a positive control for Rev-Erbα binding activity (Fig. 5a). The assay demonstrated a significant enrichment (*p* < 0.05) for Rev-Erbα binding at the *Dnmt3a* promoter (Fig. 5a). Furthermore, we transfected primary cortical neurons with specific small interfering RNAs (siRNAs) against Rev-ERBα to assess the transcriptional response of DNA methylation and demethylation enzymes in vitro. The Rev-Erbα expression was downregulated by approximately 50% (*p* < 0.0001) (Fig. 5b). We found that *Tet1* mRNA levels was significantly increased after suppression of Rev-ERBα expression, suggesting a direct inhibitory role of this transcription factor (Fig. 5b). To further test the involvement of Rev-ERBα in the control of epigenetic modifications, we conducted in vitro drug treatment experiments using a Rev-ERBα agonist (GSK 4112) [33]. Agonist treatment at 10 mM significantly downregulated Tet1 (*p* = 0.0293) and Dnmt3a (*p* = 0.0180) expression, providing a further indication that Rev-ERBα plays a repressive role in an unperturbed system (Fig. 5c).

All together, these combined data suggest that Rev-ERBα is able to interact with the transcription of DNA methylation and demethylation genes in neurons.

## Discussion

Overall, our study has shown that *Afh* mutant neuronal networks present deficiencies in global electrical activity and cell–cell functional coupling. We showed an impairment of the neuronal network in the ability to respond to external stimuli. Furthermore, the variability of signal propagation between MBL and follower loci suggested that the *Afh/Afh* neuronal networks has reduced signal transduction and therefore impaired functional coupling. Our results showed that these effects were partially reversed after treatment with Dexa, the drug used to synchronize molecular clock genes both in vivo and in vitro [18, 34–37]. Ex vivo electrophysiology using organotypic slices of *Afh* animals has previously shown how the *Fbxl3* mutation is able to prolong SCN hyperpolarization at dark–light transitions through altered GABAergic synaptic transmission [38]. Our results expand on this knowledge, providing evidence that *Afh’s* network electrophysiological properties impact on neuronal communication. Mathematical modelling of the molecular clock with neuronal coupling indicates that the *Afh* neuronal network necessitates an external input to overcome the network-disruptive effects of the *Afh* mutation. Although the role of Fbxl3 has been previously described in CRY1 degradation, and could by itself be sufficient for determining network properties such as weaker self-sustained circadian synchronization and reduced *Per2* expression, here we investigated its interaction with different clock proteins and from an epigenetic regulatory point of view.

Melanopsin indirectly regulates the activity of the cone pathway in the overall retina response [22]. Therefore, the *Afh* reduction in the amplitude of the response of retinal cone photoreceptors to light stimuli, as observed in the fERG analysis and the impairment of melanopsin protein expression in the retina indicate an aspecific effect of the mutation on circadian entrainment, which includes also the role of epigenetic mechanisms. Our evidence of an altered response to light stimuli in *Afh* is aligned with previous findings from both our group and others [14, 16]. Impaired expression of core circadian genes (i.e., *Per1* and *Per2*) in cone photoreceptors has been observed in *Opn4* knockout animals [39]*.* Furthermore, melanopsin mutations have been associated with light-dependent behavioural disturbances, such as seasonal affective disorders, in human patients [40, 41]. We have previously demonstrated that *Afh* mice show significant deficits in behavioural adjustment to changing temporary environments at multiple timescales, reduced temporal phenotypic plasticity and sleep-related alterations [14]. Interestingly, the majority of behavioural abnormalities highlighted by Maggi et al. [14] occurred at the transition between light and darkness, suggesting that melanopsin can regulate complex circadian behaviours at key circadian times of the day. Moreover, these results sustain the hypothesis that light and/or LD cycles may have a disrupting effect on behavioural phenotype as well as on sleep physiology in *Afh* mice [14] due to altered retinal sensitivity to light. Even though melanopsin elective tissue is considered retina, we found that melanopsin expression was downregulated along the RHT terminals and, in particular, in the SCNs of *Afh* mutant animals. It is known that OPN4 expressing ipRGCs deeply innervate not only SCN but also other deep brain regions, including superior colliculus, lateral habenula and olivary pretectal nucleus [42]. OPN4 protein was recently detected also in several regions of human post mortem brains and especially membranous compartments and cytoplasmic vesicles of neurons [43]. Our data about OPN4 mRNA levels and protein localization in the SCN can be explained by a localized reduced expression in *Afh* terminal projections or accumulation of melanopsin via retrograde transport. These observations point toward a more direct involvement of melanopsin in molecular pathways correlated to light inputs processing in circadian core districts as the SCN. However, whether Opn4 mRNA expression in the SCN has any role in circadian physiology remains to be understood.

A growing amount of evidence has shown that DNA methylation is a new mechanism able to tune the phenotypic plasticity of the circadian clock in the brain, especially after alterations in environmental inputs and shifting circadian conditions [6–9]. Pyrosequencing in the SCN demonstrated daily variation in the CpG methylation level of a promoter CGI of *Opn4*, especially in correspondence with the shift from light to dark conditions. Changes in methylation levels we observed in the genomes of mice previously exposed to both different day lengths and different total amounts of light per day, but never in a circadian mutant [6, 44]. However, a genome-wide scan of the SCN methylation profile was unable to detect significant global effects of DNA methylation in this region but revealed variations in a discrete number of genes, of which only *Sept1*, *Notch3* and *Nrp2* were differentially methylated in the *Afh* mutant mice. We found transcriptional differences between ZT0 and ZT12 in genes, identified by RRBS, involved in chromatin remodelling (such as *Brd4* and *Adam11*) and transcriptional plasticity processes (such as *Cdk2ap1* and *Snx9*), at both the SCN and hypothalamic levels (data not shown) [45–48]. The absence of gross methylation differences can have different explanations: first, RRBS analysis showed great variability between samples, especially in the *Afh* genotype, which in turn can be an effect of the mutation. In addition, RRBS is not able to distinguish between methylation and hydroxymethylation (5-hmC), which is another important DNA modification that constitutes a large part of the whole-cell methylome makeup and is associated with demethylation, chromatin activation and gene transcription [49]. 5-hmC may be responsible for the observed alterations in gene expression along the RHT tracts of *Afh* mutants and may explain the reduced methylation differences observed. Moreover, the observed alterations in the expression of TET enzymes in different regions may mediate the conversion of 5-mC to 5-hmC. A third possibility involves the localized roles of chromatin-modifying enzymes. Recently, ubiquitin-related molecular mechanisms have been proposed as potential new targets linking post-translational processes to the epigenetic molecular framework for the control of the circadian cellular clock. E3 ubiquitin ligase for example is known to increase sumoylation efficiency [50], which is required by *Bmal1* for maintenance of circadian rhythms [51] and for the regulation of the interaction between DNMT3s and histone deacetylases (HDACs) [52].

Impaired expression of the enzymes involved in DNA methylation in the retina as well as in the SCN and rest of the hypothalamus are shown in the current study. Using transcriptional analysis, we found that *Dnmt1*, *Dnmt3b*, *Tet1* and *Tet2* enzyme expressions were altered in *Afh* mutants during the light/dark transition, especially at ZT0, and then decreased at ZT12. These data suggest a light-dependent regulation of the enzymes involved in DNA methylation and demethylation. Our ZT expression data are consistent with other studies in Siberian hamster and Zebrafish [53, 54]. In both models, *Dnmts* and *Tets* genes show a peak of expression in the dark phase, and a decline in the light phase, in hypothalamus and gonads. *Afh* animals presented several of these genes with differential light–dark expression, in particular *Dnmt1*, which can help to maintain stable circadian oscillations preserving DNA methylation state in key regions.

Recent evidence suggests that FBXL3, component of the SKP1-CUL1-F-box (SCF) E3 ubiquitin ligase, and the mutated target in *Afh* mutants, regulates the interaction between the circadian protein Rev-Erbα and the HDAC3 repressor complex [32]. Here, we propose a possible pathway that can link the main circadian loop with the expression of epigenetic enzymes. We showed how Rev-Erbα is able to bind to *Dnmt3a* and *Tet1* promoter sequences in primary neurons and to influence the expression of these enzymes (Fig. 5a–c). This mechanism, although observed only in vitro wt neurons*,* constitutes proof of concept. Undoubtedly, further studies are needed to define the impact of DNA methylation in the RHT terminals, SCN and retina. Additional studies are also necessary to fully unravel the mechanism by which Rev-Erbα controls expression of methylation and demethylation enzymes, including in vivo or ex vivo testing.

## Materials and methods

### Mice

The after-hours mouse colony was bred at the Italian Institute of technology (IIT). All experimental procedures were conducted with age-matched groups of female mice. Wild-type (+/+) and homozygous mutant (*Afh/Afh*) animals were group-housed in the experimental room a week before the experiments with food and water ad libitum under a 12:12 LD cycle (lights on from 8:00 to 20:00). All procedures were conducted under the Italian Policy Num. 039 licence.

### Primary neuronal culture experiments

Primary cortical neurons were cultured as previously described [55]. Multi-well plates were coated the day before culture using 0.1 mg/ml poly-D-lysine (Sigma-Aldrich) and incubated overnight in a sterile incubator at 37 °C with 5% CO_2_. Embryos were individually dissected at E17–E18 to obtain cortices. 2 ml of 0.125% trypsin (Thermo Fisher Scientific) was added to each cortex and HBSS-diluted 0.25 mg/ml DNAsi (Sigma-Aldrich) was added for 30 min at 37 °C. Trypsin digestion was blocked using 2 ml of Neurobasal medium (Gibco) containing 2% B27 supplement, 1% penicillin/streptomycin, 1% L-glutamine (Life Technologies), and 10% heat-inactivated FBS (Gibco). Cells were centrifuged for 5 min at 1200 rpm and then resuspended by pipetting in 2–3 ml of complete Neurobasal medium plus FBS. Cells debris were removed by centrifuging at 700 rpm for 7 min. Neurons were then resuspended in complete culture medium without FBS, counted with trypan blue dye (Sigma-Aldrich) and then plated at a concentration of 500,000 cells/well. For micro-electrode arrays (MEAs) electrophysiological experiments, neurons were plated at a final concentration of 36–40,000 cells/ml. Cells were plated on to 60-channel 6-well and 60-channel single-well MEAs previously coated with poly-D-lysine and laminin (Sigma-Aldrich) to promote cell adhesion (final density of approximately 1,200 cells/mm^2^), as previously reported [56, 57]. Medium was changed by half twice per week.

### Gene expression analysis in primary synchronized neuronal cultures

Gene expression profiles where computed using the 2^-DDCT method. DCT for a circadian time point was computed as the difference in CT between target gene and GAPDH house-keeping gene. DDCT was computed by subtracting the DCT of a time point with DCT computed at circadian time 6. This procedure resulted in a time series of relative expression values for each gene and sample. We fit the collection of time series for individual genes with a sinusoidal function f(t) = L + A sin(\phi + 2\pi t/T) parametrized by translation L, amplitude A, phase \Phi and period T in order to estimate the expression periodicity.

Regression results and statistics were obtained by orthogonal distance regression, using the Python library spicy.odr. Statistics includes mean, STD, confidence interval and significance *p* values value for each regression parameter.

### MEA electrophysiology

Electrical activity of 20 DIV neuronal cultures was recorded on both 6-well and single-well MEAs (Multichannel Systems, MCS; Reutlingen, Germany) consisting of 60 TiN/SiN planar round electrodes (30-μm diameter; 200-μm centre-to-centre inter-electrode distance). The activity of all cultures was recorded using the MEA60 System (MCS). Signals were first amplified 1200x, sampled at 10 kHz, and acquired through the data acquisition card and MC_Rack software (both from MCS). Thermal stress, evaporation and osmolarity were constantly monitored using a controlled thermostat (MCS), a polydimethylsiloxane (PDMS) cap and a custom chamber with controlled atmosphere, as previously reported [56]. The experimental protocol consisted of a control phase lasting 180 min during which recording was performed in Neurobasal complete medium (2% B27, 1% penicillin/streptomycin, 1% L-glutamine). Then, 100 nM dexamethasone (Sigma-Aldrich) was added to the cultures by direct pipetting into the medium. Time 0 was set at 24 h after treatment. First ten minutes of recording were discarded to avoid perturbation of the firing rate. The total number of experiments performed by following the above protocol (i.e., the number of recorded wells) was as follows: 24 h recording without treatment, +/+ * n* = 5, number of embryos = 4, and *Afh/Afh* *n* = 5, number of embryos = 5; and 24 h recording with treatment, +/+ * n* = 5, number of embryos = 4, and *Afh/Afh* *n* = 5, number of embryos = 5. The data were high-pass-filtered at 300 Hz with the online software MC_Rack (MCS) to selectively consider multiunit activity (MUA) only. Spikes were detected online using a fixed threshold multiple of the standard deviation of the noise (-5σn). The data analysis was performed using offline custom software developed in MATLAB (MathWorks) called SPYCODE that presents a number of different tools suitable for multichannel neural recording [58]. Cortical neuronal cultures presented two patterns of activity: random spikes and bursts. The bursting behaviour was characterized by a highly dense packed of spikes usually occurring simultaneously in many channels. The burst detection method was used as previously described [59]. After the identification of spikes and bursts, we analyzed several parameters describing the electrophysiological patterns, such as the firing rate (the mean number of spikes per second calculated on the active channels). Moreover, we analyzed the number of synchronized bursts, called network bursts (NBs), in all active channels using a custom algorithm [57].

The recent studies have found that the majority of NBs are led by a small group of cells called major burst leaders [60], which are defined as neurons that have a probability of conducting more than 6% of the total number of detected NBs. To quantify how the NBs propagate within the network, we calculated the minimum propagation delay between each detected MBL and the other electrodes, called followers [61]. We considered the difference between the delay of the first follower and that of the last follower (Fig. 3d). Finally, we analyzed the spike train correlation among 60 channels. The cross-correlation function represents the probability of observing a spike in one channel *i* at time *t* + *τ* (*τ* = 3 ms) given that there is a spike in a second channel *i* + *1* at time *t*. We considered only the channels with a peak of correlation higher than 0.1. To quantify the changes in the synchronicity, we evaluated the coincidence index, which represents the ratio between the cross-correlation area around zero (± 3 ms) and the total area. If the data had normality and equal variance, a two-way ANOVA was used with Tukey post-test, while if not, data were ranked and then analyzed by ANOVA with post-test.

### Circadian clock simulation

We implemented mathematical simulations of the circadian loop as described in a stochastic model of the circadian clock in neurons [21]. All reaction rates and parameters were set as in [21], with the exception of the CRY1 degradation rate, which was modulated to mimic the lengthening of circadian periods due to the after-hours mutation. The code used for all simulations was written in the C-programming language and consisted of a modified version of the Gillespie algorithm [21] with the addition of a random seed generator used for multiple simulations.

In each run of the algorithm, we simulated a population of 100 neurons for 16 days, and we recorded the PER2 levels with a 5-min resolution. For each cell, we extracted PER2 periodicity by fitting the expression traces with a sinusoidal function using the “optimize.curve_fit” function from the Python package SciPy.

To extract the CRY1 degradation rate corresponding to the after-hours mutation, we performed a number of simulations decreasing the degradation rate starting from the wild-type scenario indicated by Ko et al. [21]. Higher degradation rates resulted in shorter circadian periodicity with an exponentially decaying profile. The in silico results were fitted with an exponential ordinary least squares regression model. The resulting exponential equation was used to find the degradation rate that reproduced the after-hours circadian period (approximately 26.7 h [12]).

### Flash electroretinogram (fERG) assay

 +/+ (*n* = *14*) and *Afh/Afh* (*n* = 14) mice were used. Mice were kept in constant darkness for 1 h before midday (12:12 LD cycle) as previously reported [22] and then anesthetized with urethane by intraperitoneal (IP) injection (Sigma-Aldrich). Mice were gently restrained using a mouth bar on an electrophysiological stage as described previously [62]. After adding a few drops of Tropicamide 1% reagent (VISUfarma) to the eyes to dilate pupils, mice were kept in the dark for 10 min. The recording and ground electrodes were then mounted. Visual stimuli, consisting of uniform flickers of light of different luminance values (0.003, 0.01, 0.1, 1, 10 and 30 cd s/m^2^), were applied using a scotopic background on a Ganzfeld dome (Bioptica Mangoni). To saturate rod response and measure cone activity, fERG was measured in photopic conditions applying a background of 20 cd s/m^2^. After 5 min of background illumination, a single flash with a luminance of 30 cd s/m^2^ was applied. Retinas were harvested at the end of the experiment. The amplitude and implicit responses of the b wave were measured on the electroretinographs.

### Western blotting

Two groups of +/+ (*n* = 13) and *Afh/Afh* (*n* = 11) animals were sacrificed by dislocation for biochemical analysis at ZT0 (*n* = 7 and *n* = 6, respectively) and ZT12 (*n* = 6 and *n* = 5, respectively). For protein extraction from the retina, the eyecups were removed, and the retinas were dissected in ice-cold PBS and snap frozen on dry ice. Retinas were lysed by sonication in 150 mM NaCl, 10 mM Tris pH 7.4, 1 mM EGTA, 0.5% Triton X-100 (Sigma-Aldrich), with protease and phosphatase inhibitor cocktail (Roche) and incubated for 30 min on ice. Samples were centrifuged at 20,800 × *g* for 20 min at 4 °C. Supernatants were collected, and protein concentrations were determined using a BCA kit (Pierce) following the manufacturer’s instructions. Equal amounts of proteins were run and separated by 10% sodium dodecyl sulphate polyacrylamide gel electrophoresis (SDS-PAGE) as previously described [63]. Proteins were transferred overnight at 4 °C onto nitrocellulose membranes (GE Healthcare). Membranes were blocked with 5% semi-skimmed milk in PBS (pH 7.4) containing 0.05% Tween 20 (Sigma-Aldrich). Primary antibody incubation was performed overnight at 4 °C using an anti-Melanopsin antibody (rabbit anti-Melanopsin, 1:500, #PA1-780, Thermo Fisher Scientific) in blocking buffer. Membranes were then rinsed at least three times in Tris-buffered saline (pH 7.4) with 0.05% Tween 20 (TBST) and then incubated for 1 h at room temperature with horseradish peroxidase (HRP)-conjugated secondary antibodies (Immunopure peroxidase-conjugated, Thermo Fisher Scientific). Proteins were visualized using Immobilon Western Chemiluminescence Kit (Millipore). Chemiluminescence signals were acquired using an Image Quant LAS 4000 Mini apparatus (GE Healthcare) and densitometric analysis was performed using NIH ImageJ Software [64]. Protein levels are expressed as ratios with respect to β-tubulin levels. The glycosylated OPN4 form was normalized to the level of the unglycosylated form.

### RT-qPCR analysis

Transcriptional analysis of the indicated genes was performed using another independent set of 13-week-old +/+ and *Afh/Afh* animals. For the genotype analysis, a cohort of animals of each genotype was sacrificed throughout the circadian day. For time point investigation, one set of mice was culled at ZT0 (at the beginning of the light phase, immediately after the light onset), and one was culled at ZT12 (at the beginning of the dark phase, immediately after the light offset). All tissues were rapidly dissected using a tissue puncher on an ice-cold surface and frozen on dry ice. For the SCNs, experiments were run to produce data from *n* = 24 +/+ and *n* = 23 *Afh/Afh* mice for genotype experiments and for *n* = 10 per genotype for the two time points. The prefrontal cortex and the rest of the hypothalamus were harvested as reported above (*n* = 12 for each region and genotype). The eyes were enucleated from 8 mice per genotype, and retinas were rapidly dissected and placed in liquid nitrogen. For the SCNs, total RNA was extracted from snap-frozen tissue using a RNeasy Tissue Micro Kit (Qiagen) and QIAzol reagent (Qiagen) in combination with a Tissue-Lyser apparatus (Qiagen) following the manufacturer’s instructions. RNA samples were quantified with an ND1000 NanoDrop spectrophotometer (Thermo Fisher Scientific). RNA from the retina, prefrontal cortex and hypothalamus was purified and treated as described previously [65]. Reverse transcription of approximately 800 ng of RNA was performed using an ImpromII Reverse Transcription Kit (Promega) according to the manufacturer’s instructions. RT-qPCR was performed using an ABI Prism 7900 RT-qPCR machine (Applied Biosystems) and SYBR Green master mix (Qiagen). The reactions were carried out in triplicate. The data analysis was performed as previously described with minor modifications [65]. All samples were normalized on a panel of three housekeeping genes: *Gapdh, ß-actin* and *Hprt1*. The expression levels relative to these housekeeping genes were determined by calculation of the ΔCt, and the data are expressed as 2^−ΔΔCt^, where ΔΔCt is the difference between the +/+ ZT0 cohort and the other experimental cohorts for the time point experiments and between the +/+ pooled samples and the other samples for the genotype analyses. For in vitro experiments, neurons were washed three times with ice-cold phosphate-buffered saline (PBS) solution and lysed with 350 μl of RNeasy Lysis Buffer (Qiagen) or 300 μl of TRIzol (Life Technologies). Cell lysates were collected and pipetted several times through a 22-gauge needle into 1.5-ml microcentrifuge tubes to better lyse the cells, and the lysates were stored at − 80 °C until RNA extraction. RNA was isolated using a RNeasy Micro Kit (Qiagen) according to the manufacturer’s protocol. cDNA synthesis was performed with an ImpromII Reverse Transcription Kit (Promega) according to the manufacturer’s specifications. Real-time PCR was performed and analyzed as above with at least duplicate wells for each sample at each time point. All the primers used in real-time PCR experiments both ex vivo and in vitro are reported in Additional file 12: Table S6.

### Reduced-representation bisulphite sequencing (RRBS)

Thirteen-week-old female +/+ and *Afh/Afh* mice were euthanized by dislocation. At least two for each genotype were euthanized at zeitgeber time (ZT) 0 (at the beginning of the light phase, immediately after the light onset) and at ZT12 (at the beginning of the dark phase, immediately after the light offset). SCNs were rapidly dissected using a tissue puncher on an ice-cold surface and frozen in dry ice. SCN tissue punches were validated for region specificity (Additional file 6: Figure S5) using the specific marker gene *Six6*, which is selectively expressed in the SCNs of adult rodents [66]. The dissected SCNs were used for DNA genomic methylation screening by RRBS and for pyrosequencing assays. Genomic DNA was extracted and purified using a QIAamp DNA Micro kit (Qiagen) following the manufacturer’s instructions. Purified DNA was quantified using an ND1000 NanoDrop spectrophotometer from Thermo Fisher Scientific, and 100 ng was used for RRBS library generation as previously reported with minor modifications [27, 67, 68]. The bisulphite-converted DNA was indexed using KAPA U polymerase (KAPA Biosystems) and RRBS Multiplex TAG primers (10 μM, Sigma-Aldrich). The processed DNA was purified using solid-phase reversible immobilization (SPRI) beads (Agencourt) following the manufacturer’s specifications. A second round of amplification (15 cycles) was performed using KAPA U uracil stalling-free polymerase (KAPA Biosystems). An additional SPRI purification step was performed, and the sample libraries were screened with a Bionalyzer (Agilent Technologies) and then sequenced on an Illumina HiSeq 2500 platform (Illumina). All the primers and probes used for RRBS sample generation are shown in Additional file 13: Table S2. Because of the low complexity at the start of each sequence (MspI fragments), dark cycles were performed (the first 4 bases of each sequence were not recorded). Sequence alignment and methylation calling of the RRBS datasets were performed using Bismark software [69]. CpGs with read depths < 5 were discarded. For every analysis, all informative CpGs were used. Mapping efficiency across the two genotypes was high (64.03% ± 0.38 and 60.87% ± 0.57 for *Afh/Afh* and +/+ mice, respectively; see Table S3. Moreover, the CpG methylation was 23.3% ± 0.65 in *Afh/Afh* mice and 22.3% (± 0.65) in +/+ mice, confirming coverage levels previously reported at CpG dinucleotides in the mammalian genome [25, 26, 70, 71]. Indeed, the CHG and CHH methylation levels (where H is A, C or T) were ~ 1% in both groups (Additional file 8: Table S3).

To score CpG island (CGI) methylation, cutoffs were applied. The methylation levels were determined for CGIs with information on ≥ 10% of their total CpGs (with a minimum of 3 CpGs) and by averaging individual cytosine methylation levels.

Logistic regression was further applied to identify differentially methylated CGIs using a *p* < 0.05 after correction for multiple comparisons with the Benjamini–Hochberg procedure. A cutoff of a minimum methylation difference of 10% between groups was also used. In addition, a replicate test (*t* test/ANOVA) (*p* < 0.01) was applied to identify the strongest candidates from the hits obtained by logistic regression.

The dataset analysis was based on the Grcm38 build of the mouse genome and was performed using the SeqMonk software suite. Promoter CGIs were defined as overlapping an annotated transcription start site (TSS), using the University of California, Santa Cruz (UCSC) or Ensemble databases. Intragenic CGIs were defined as overlapping an annotated gene without its TSS. Promoters were defined as the region 2 kb upstream of annotated TSSs. Among the 31 differentially methylated CGIs in *Afh/Afh* mice, six mapped to intergenic regions and were annotated as ‘null’.

### Pyrosequencing

Snap-frozen SCN tissue was treated as previously reported, and 100 ng of genomic DNA was bisulphite converted using an Imprint DNA Modification kit (Sigma-Aldrich, USA) following the manufacturer’s instructions. Pyrosequencing analysis was conducted by NXT-DX (Belgium), as previously reported [72]. The sequences of the primers used in this study are as follows: Opn4F, TTAGTGTGGTTGTTGAGTTG, biotinylation modified; Opn4R, AAAACTTTAAAAATATTCCTATCAC; and Opn4S2, AAAATATTCCTATCACTC.

### Immunofluorescence of SCN slices

Wild-type and mutant mice were sacrificed by cardiac perfusion. Animals were anaesthetized using IP injection of 20% urethane solution and were then transcardially perfused with 4% freshly prepared PFA (Sigma-Aldrich) in 0.1 M phosphate buffer (PB; pH 7.4). Brains were extracted, placed in a 2% PFA solution for approximately 2 h and then transferred to a 30% sucrose solution overnight to allow cryopreservation of the brain structures and morphology. Brains were rinsed several times in 0.1 M PBS solution and then cut coronally (50-μm thick slices) using a Microm KS34 freezing microtome (Thermo Fisher Scientific). Free-floating slices were collected in serial order throughout the entire hypothalamic region, considering that the mouse SCNs are between − 0.22 and − 0.82 μm from the Bregma.

Slices were permeabilized and blocked using 3% normal goat serum (NGS) and then 0.3% Triton X-100 in PBS for 2 h. Slices were incubated with OPN4 antibodies overnight at 4 °C (PA1-780, Thermo Scientific, 1/100 primary, 1/500 secondary). Cell nuclei were detected using DAPI diluted 1:300 in PBS for 10 min at room temperature. Images were acquired using an Eclipse Ti A1 confocal inverted microscope (Nikon, Japan). Acquisitions were automatically performed using the motorized Z-stack function of the A1 microscope.

### Bioinformatics analysis of transcription factor binding sites (TFBSs)

Gene names of targets of interest from the RRBS screening and of enzymes involved in methylation pathways were imported into the Genomatix software program as previously described [73, 74]. Promoter sequences of the genes were then identified and retrieved using LASAGNA 2.0 software [75]. The promoter regions were further analyzed for common TFBSs using the MatInspector suite of Genomatix [73].

### Chromatin immunoprecipitation (ChIP)

Primary cortical neurons from E17–E18 +/+ embryos were cultured in Neurobasal complete medium supplemented with L-glutamine, penicillin/streptomycin and B27 supplement. Cells were harvested and processed for ChIP assays using a LowCell ChIP kit (Diagenode) according to the manufacturer’s instructions. Chromatin shearing was performed using Bioruptor Plus (Diagenode) for 10 cycles of 20 s on/20 s off. Sheared chromatin equivalent to 100,000 cells was used for immunoprecipitation using 8 µg of anti-Rev-ERBα antibody (13,418 Cell Signaling), and normal rabbit IgG (Sigma-Aldrich) was used as a negative control. The isolated DNA samples were analyzed by RT-qPCR using promoter primers for the following epigenetic targets: *dnmt3a* (first pair, Fw AACGGTGTCCTTGTCCTC and Rv ATTTCTGCCACCCATAGTCT*;* second pair, Fw AGGTCTAGTGCCCGTCTG and Rv TGAAGAGGTGGAAGGTTGAAC), *dnmt3b* (Fw GAGGAACCCAGGTAGTTG and Rv TTCTGCTTCCTGCTTTCA), *Tet1* (Fw GCTATTGTTATTTTAGACCCCAAA and Rv ATCTTCCTTTTGAGGAGAATCTG), and *Bmal1* (Fw AGCGGATTGGTCGGAAAGT and Rv ACCTCCGTCCCTGACCTACT). The data were normalized to the input and plotted as the % of recovery.

### Small interfering RNA (siRNA) experiments

Neurons were obtained from +/+ embryos at E17-E18. Cortical neurons at 6 DIV were transfected with Silencer Select Pre-designed siRNA against Rev-ERBɑ (siRNA ID#: s117137) or with control siRNA (Life Technologies) using Lipofectamine RNAiMAX Transfection Reagent (Life Technologies). Neurons were seeded in 6-well plates at a density of 600,000 cells/well. siRNAs were used at 10 μM concentrations according to the manufacturer specifications, as previously reported [55]. Neurons were harvested at 8 DIV and analyzed for gene expression of epigenetic targets.

### Drug treatments

Neurons were collected from wild-type littermate embryos at E17–E18 and plated in 12-well plates at a final density of 500,000 cells per well. Neurons were treated with 10 μM GSK 4112 (Sigma-Aldrich) or DMSO in normal Neurobasal complete medium as a control for 24 h. The drug was then removed, and the cells were harvested to investigate the gene expression profiles of DNA methylation genes.

### Statistical analysis

The data were analyzed with Microsoft Excel, Prism (GraphPad), Sigma Plot and Python software. Biochemistry and genomics experiments were processed in parallel simultaneously. Statistical comparison of differences among groups of data was carried out using Student’s *t* test. For comparison of more than two groups, two-way ANOVA was used with Bonferroni post hoc tests. Significant differences were considered as follows: * p < 0.05, ** p < 0.005 and *** p < 0.001.

## Conclusion

In conclusion, our electrophysiological and molecular phenotypic data constitute a preliminary insight into the complexity of the epigenetic landscape of the circadian control by Fbxl3.

## Supplementary information

**Additional file 1: Figure S1**. In vitro synchronization of core clock genes using Dexamethasone. (A) Clock genes mRNA expression of wt primary neurons after synchronization with dexamethasone. Error bars are mean ± standard deviation of 2^DDCT, where the normalization is computed with respect to CT6. Gene expression time courses were fit with the sinusoid f(t) = L + A sin(\phi + 2 \pi /T t) in order to extract periodicity, phase and amplitude of the oscillation. Sinusoidal profiles are overlapped to the expression levels in the figures, fits statistics are reported in Additional file 2: Table S1.

**Additional file 2: Table S1.** Fitting parameters of in vitro syncronized circadian genes.

**Additional file 3: Figure S2**. In silico modelling confirms and reproduces *Afh* circadian circuit abnormalities. (A) Schematic representation of the reaction for the stochastic model of the circadian clock in SCN neurons with neuronal coupling (figure modified from [21]). (B) Identification of a candidate degradation rate for *Afh/Afh* simulations. The circadian clocks of individual cells were simulated for a population of 100 neurons for different rates of CRY1 degradation (k_degr_ values). The *x* axis shows the fraction of k_degr_ with respect to the wild-type degradation rate taken from [21]. The *y* axis shows the periodicity of PER2 levels in hours. The blue dots represent the PER2 periodicity of the in silico neuronal population averaged over three repetitions of the circadian clock simulation. The red curve represents an exponentially decaying curve regressed over the in silico results. The black dashed line shows the PER2 periodicity levels of *Afh/Afh* mice from [12]. The intersection between the red and black lines represents our choice for the k_degr_ for *Afh/Afh* mice, which was 34% of the wild-type k_degr_. (C) +/+ (black line) and *Afh/Afh* (red line) population PER2 levels normalized to the first time point of wild-type simulation. (D) Histogram of single-cell periodicity extraction. The PER2 periodicities of individual neurons were extracted with a sinusoidal fit and are reported in a histogram representing the probability density of periods over the in silico population. (E) Violin plot representing the average expression levels of *Per2* from the in silico population. (F) Violin plot from RT-qPCR of the synchronized primary neuronal cell culture. The wild-type expression levels are reported in grey, while the red violins represent the *Afh/Afh* expression levels. * *p* < 0.05. Statistical analysis in E–F was performed using Student’s *t* test.

**Additional file 4: Figure S3**. OPN4 and methylation enzymes levels are differentially expressed in several brain areas of *Afh/Afh* animals between zt0 and zt12. (A) Relative expression of OPN4 in the Retina normalized to multiple housekeeping genes at the two different time points, ZT0 and ZT12. (B) RT-qPCR of methylation-mediating enzymes in the Hypothalamus at the two different time points, ZT0 and ZT12. (C) RT-qPCR of methylation-mediating enzymes in the Retina at the two different time points, ZT0 and ZT12. All expression levels in A–C are normalized to those of multiple housekeeping genes. All bars represent the average ± SEM for at least two different experiments (minimum *n* = 3 for each genotype at each time point). * *p* < 0.05, *** *p* < 0.001, two-way ANOVA plus Bonferroni post-test.

**Additional file 5: Figure S4**. *Afh/Afh* show alteration in OPN4 protein levels during the light–dark transitions in the Retina. (A) Western blot of Opn4 in retina of Afh/Afh (*n* = 11) and +/+ (* n* = 13). (B) Histogram representing OPN4 53 KDa level at the two different time points ZT0 and ZT12. (C) Histogram showing OPN4 85 KDa levels at the two different ZT. (D) Representative 40 × images of 30 µM slices from *Afh/Afh* mice immunoblotted with anti OPN4. OPN4 expression was detected in both nuclei and fibres. DAPI was used as a nuclear marker. * *p* < 0.05, two-way ANOVA test with Bonferroni post hoc tests (panels B–C). All image analyses were performed with ImageJ software.

**Additional file 6: Figure S5**. Dissected SCN showed specific marker expression. (A) SCN dissection validation using RT-qPCR of a specific SCN marker gene *Six6* in SCN, striatum and hippocampus (*n* = 3 per tissue). (B) *Six6* expression levels in *Afh/Afh* and +/+ SCN samples (*n* = 3 per genotype). (A–B) The bars represent the average values ± SEMs. *** *p* < 0.001, one-way ANOVA plus Bonferroni post-test.

**Additional file 7: Figure S6**. Reduced-representation bisulphite sequencing (RRBS) genomic coverage. (A) Total CpG coverage on a genome-wide scale. The percentage of CpGs sequenced with more than 1, 3 and 5 reads per sequence for each *Afh* animal is plotted. (B) Percentages of CpGs covering and mapping to known CGIs. The percentage of CpGs in CpG islands (CGIs) with more than 1, 3 and 5 reads per sequence for each *Afh* animal is plotted.

**Additional file 8: Table S3.** RRBS libraries statistics.

**Additional file 9: Table S4.** CpG and CGI coverage of RRBS dataset.

**Additional file 10: Table S5.** Localization of RRBS identified target regions.

**Additional file 11: Table S7.** Math inspector table of ROR family binding sites.

**Additional file 12: Table S6.** RT-qPCR primer sequences.

**Additional file 13: Table S2.** RRBS primer sequences.

## Data Availability

The datasets used and/or analyzed during the current study are available from the corresponding author on reasonable request.
