# Supplementary figures and images for: Cell–cell coupling and DNA methylation abnormal phenotypes in the after-hours mice
Source: Epigenetics Chromatin. 2021 Jan 6;14:1. doi: 10.1186/s13072-020-00373-5 (PMC7789812; doi:10.1186/s13072-020-00373-5)

FIGURE S1

A

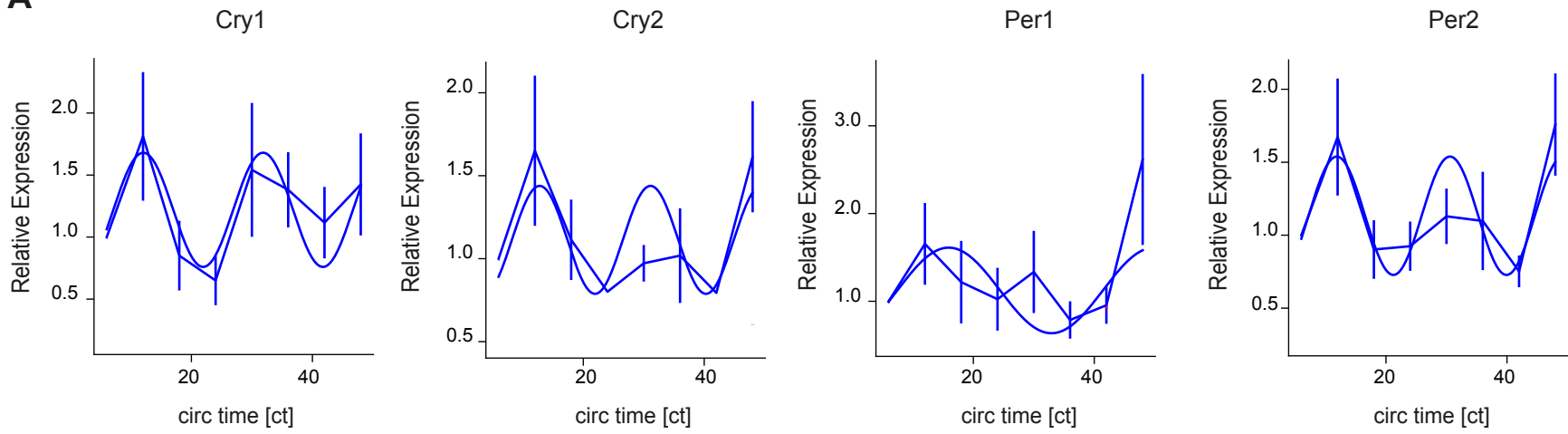

Supplement: Supplementary file 1 — Additional file 1: Figure S1. In vitro synchronization of core clock genes using Dexamethasone. (A) Clock genes mRNA expression of wt primary neurons after synchronization with dexamethasone. Error bars are mean ± standard deviation of 2^DDCT, where the normalization is computed with respect to CT6. Gene expression time courses were fit with the sinusoid f(t) = L + A sin(\phi + 2 \pi /T t) in order to extract periodicity, phase and amplitude of the oscillation. Sinusoidal profiles are overlapped to the expression levels in the figures, fits statistics are reported in Additional file 2: Table S1. [file 13072_2020_373_MOESM1_ESM.pdf]

**FIGURE S2****A**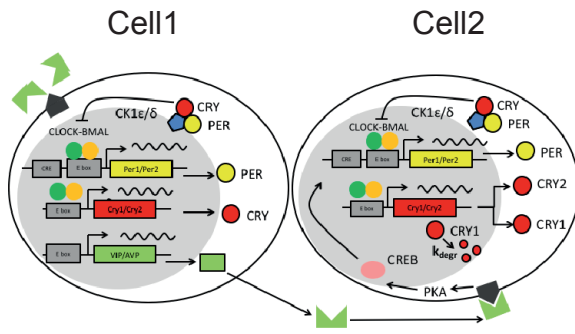**B**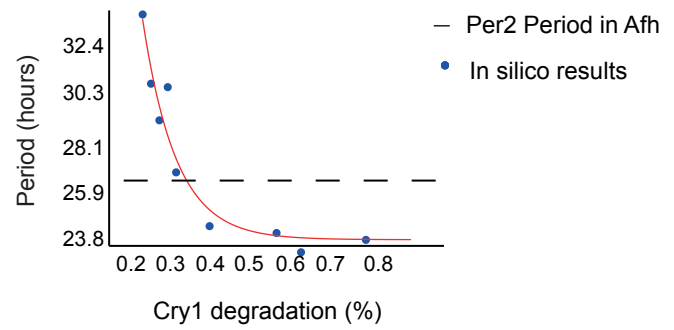**C**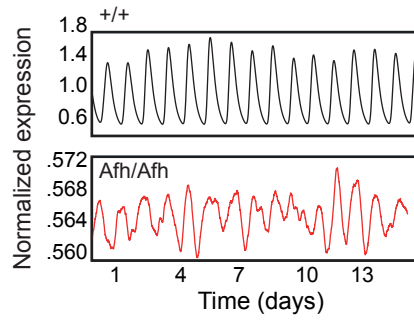**D**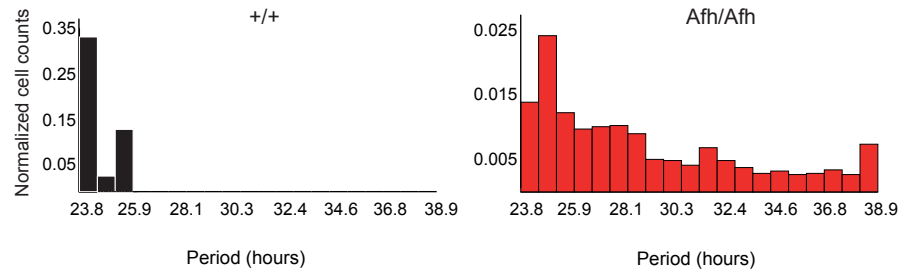**E**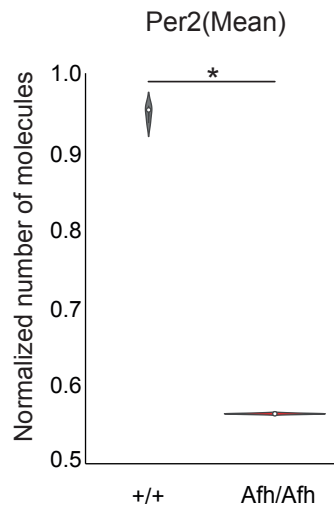**F**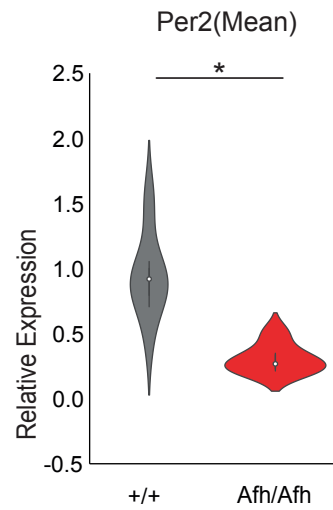

Supplement: Supplementary file 3 — Additional file 3: Figure S2. In silico modelling confirms and reproduces Afh circadian circuit abnormalities. (A) Schematic representation of the reaction for the stochastic model of the circadian clock in SCN neurons with neuronal coupling (figure modified from [21]). (B) Identification of a candidate degradation rate for Afh/Afh simulations. The circadian clocks of individual cells were simulated for a population of 100 neurons for different rates of CRY1 degradation (kdegr values). The x axis shows the fraction of kdegr with respect to the wild-type degradation rate taken from [21]. The y axis shows the periodicity of PER2 levels in hours. The blue dots represent the PER2 periodicity of the in silico neuronal population averaged over three repetitions of the circadian clock simulation. The red curve represents an exponentially decaying curve regressed over the in silico results. The black dashed line shows the PER2 periodicity levels of Afh/Afh mice from [12]. The intersection between the red and black lines represents our choice for the kdegr for Afh/Afh mice, which was 34% of the wild-type kdegr. (C) +/+ (black line) and Afh/Afh (red line) population PER2 levels normalized to the first time point of wild-type simulation. (D) Histogram of single-cell periodicity extraction. The PER2 periodicities of individual neurons were extracted with a sinusoidal fit and are reported in a histogram representing the probability density of periods over the in silico population. (E) Violin plot representing the average expression levels of Per2 from the in silico population. (F) Violin plot from RT-qPCR of the synchronized primary neuronal cell culture. The wild-type expression levels are reported in grey, while the red violins represent the Afh/Afh expression levels. * p < 0.05. Statistical analysis in E–F was performed using Student’s t test. [file 13072_2020_373_MOESM3_ESM.pdf]

**FIGURE S4**

**A**

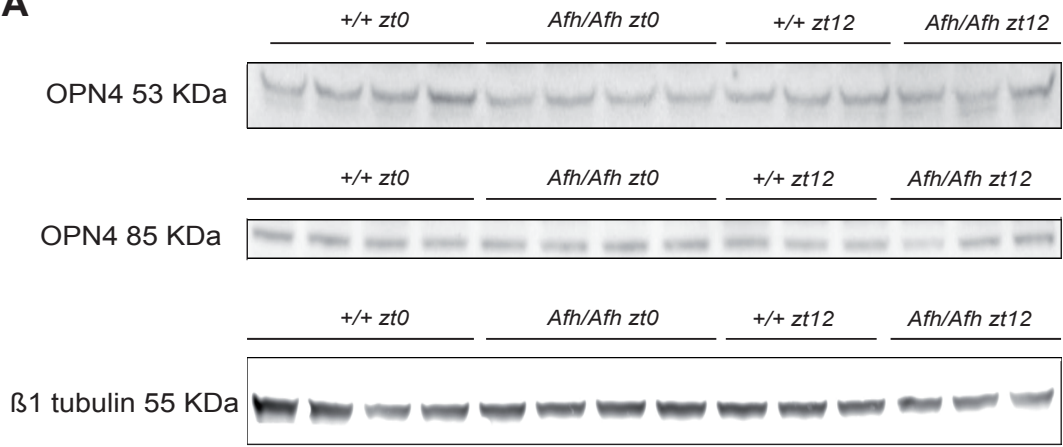

**B**

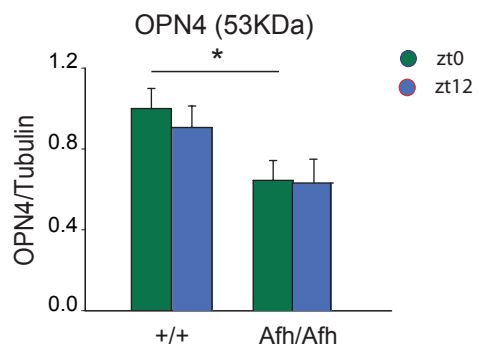

**C**

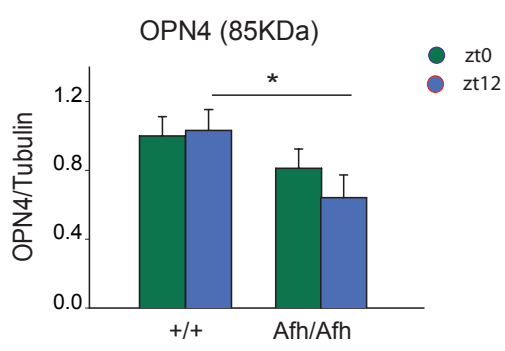

**D**

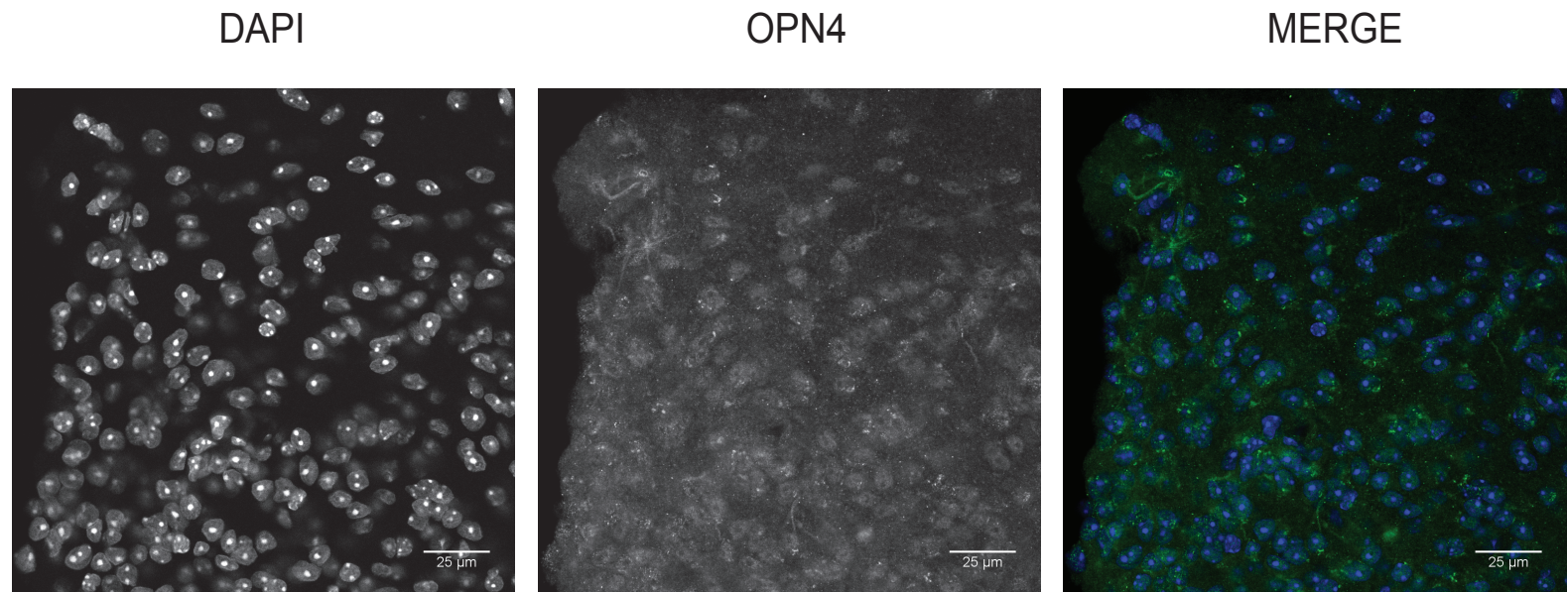

Supplement: Supplementary file 4 — Additional file 4: Figure S3. OPN4 and methylation enzymes levels are differentially expressed in several brain areas of Afh/Afh animals between zt0 and zt12. (A) Relative expression of OPN4 in the Retina normalized to multiple housekeeping genes at the two different time points, ZT0 and ZT12. (B) RT-qPCR of methylation-mediating enzymes in the Hypothalamus at the two different time points, ZT0 and ZT12. (C) RT-qPCR of methylation-mediating enzymes in the Retina at the two different time points, ZT0 and ZT12. All expression levels in A–C are normalized to those of multiple housekeeping genes. All bars represent the average ± SEM for at least two different experiments (minimum n = 3 for each genotype at each time point). * p < 0.05, *** p < 0.001, two-way ANOVA plus Bonferroni post-test. [file 13072_2020_373_MOESM4_ESM.pdf]

FIGURE S3

A

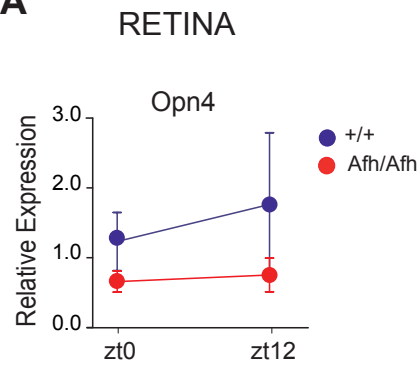

B

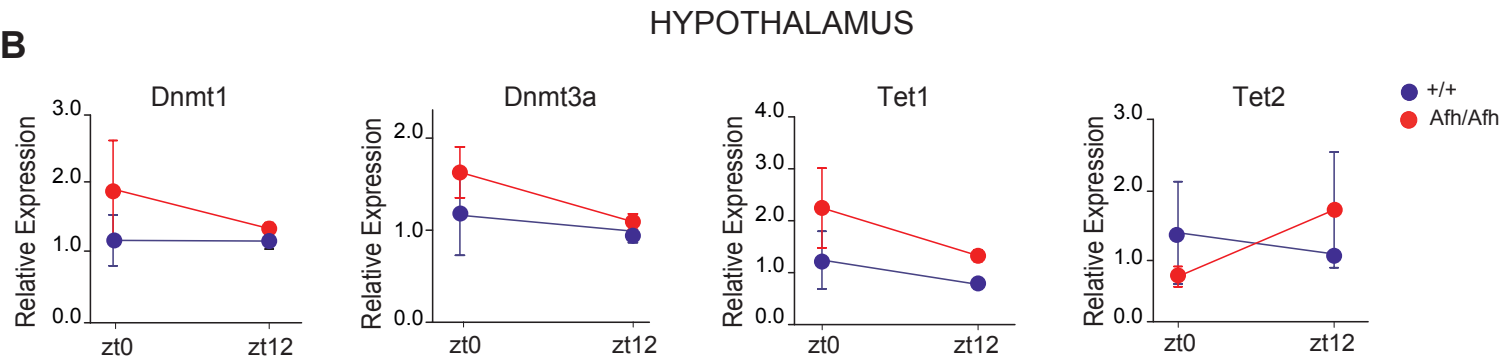

C

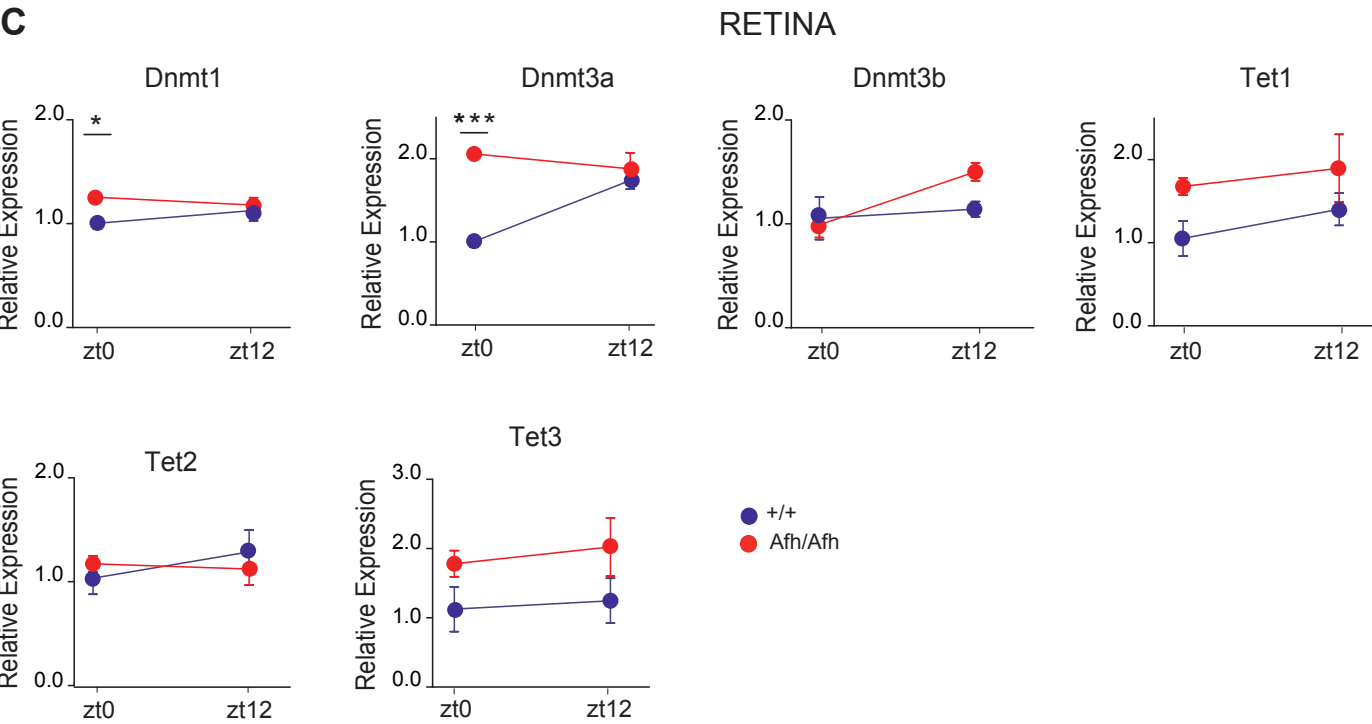

Supplement: Supplementary file 5 — Additional file 5: Figure S4. Afh/Afh show alteration in OPN4 protein levels during the light–dark transitions in the Retina. (A) Western blot of Opn4 in retina of Afh/Afh (n = 11) and +/+ ( n = 13). (B) Histogram representing OPN4 53 KDa level at the two different time points ZT0 and ZT12. (C) Histogram showing OPN4 85 KDa levels at the two different ZT. (D) Representative 40 × images of 30 µM slices from Afh/Afh mice immunoblotted with anti OPN4. OPN4 expression was detected in both nuclei and fibres. DAPI was used as a nuclear marker. * p < 0.05, two-way ANOVA test with Bonferroni post hoc tests (panels B–C). All image analyses were performed with ImageJ software. [file 13072_2020_373_MOESM5_ESM.pdf]

FIGURE S5

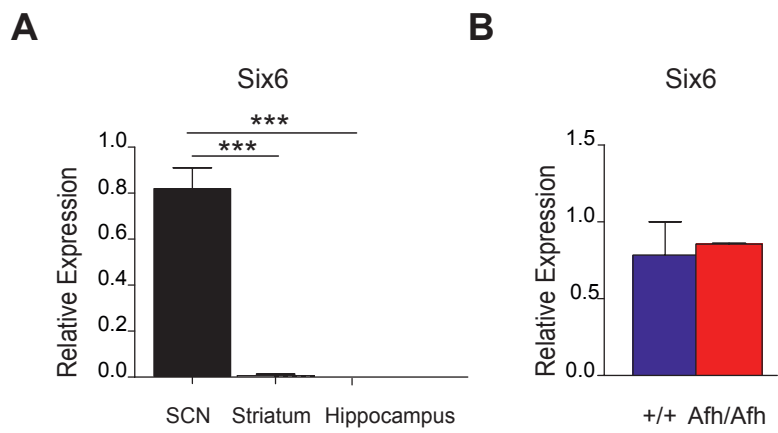

Supplement: Supplementary file 6 — Additional file 6: Figure S5. Dissected SCN showed specific marker expression. (A) SCN dissection validation using RT-qPCR of a specific SCN marker gene Six6 in SCN, striatum and hippocampus (n = 3 per tissue). (B) Six6 expression levels in Afh/Afh and +/+ SCN samples (n = 3 per genotype). (A–B) The bars represent the average values ± SEMs. *** p < 0.001, one-way ANOVA plus Bonferroni post-test. [file 13072_2020_373_MOESM6_ESM.pdf]

FIGURE S6

A

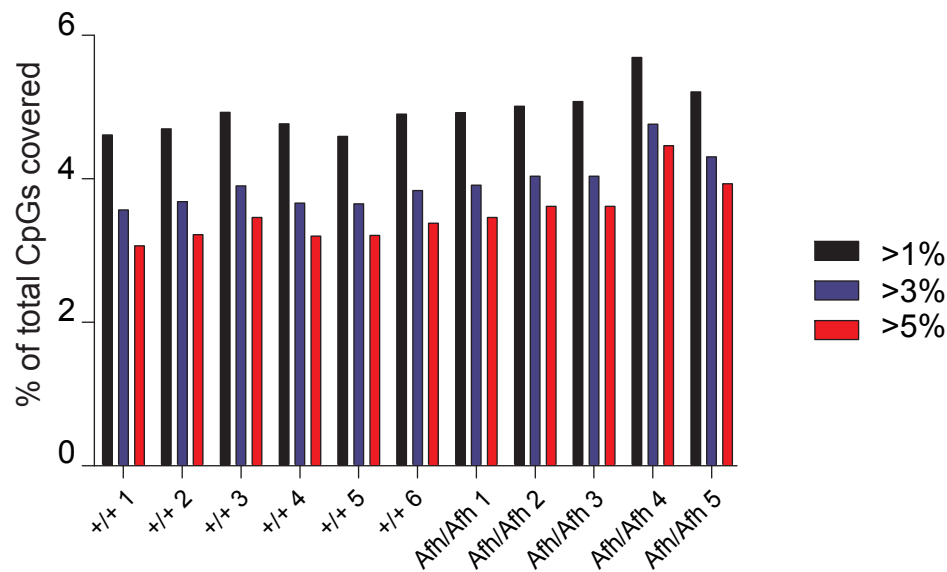

B

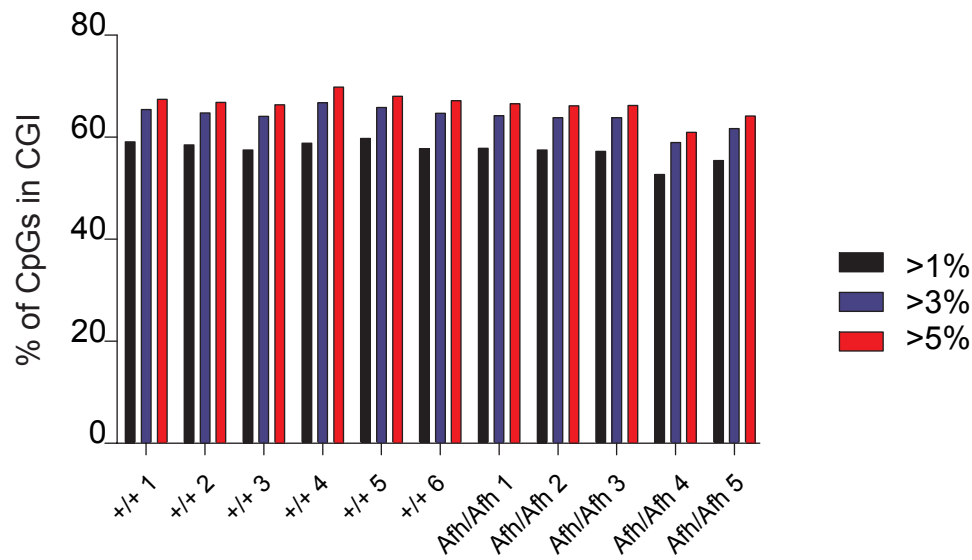

Supplement: Supplementary file 7 — Additional file 7: Figure S6. Reduced-representation bisulphite sequencing (RRBS) genomic coverage. (A) Total CpG coverage on a genome-wide scale. The percentage of CpGs sequenced with more than 1, 3 and 5 reads per sequence for each Afh animal is plotted. (B) Percentages of CpGs covering and mapping to known CGIs. The percentage of CpGs in CpG islands (CGIs) with more than 1, 3 and 5 reads per sequence for each Afh animal is plotted. [file 13072_2020_373_MOESM7_ESM.pdf]
