# Supplementary material for: Cell–cell coupling and DNA methylation abnormal phenotypes in the after-hours mice
Source: Epigenetics Chromatin. 2021 Jan 6;14:1. doi: 10.1186/s13072-020-00373-5 (PMC7789812; doi:10.1186/s13072-020-00373-5)
Supplement: Supplementary file 10 — Additional file 10: Table S5. Localization of RRBS identified target regions. [file 13072_2020_373_MOESM10_ESM.docx]

**Additional file 10: Table S5: Math Inspector Genomatix TFBS results**

| Gene symbol | Family/Matrix | Opt. | Position | Strand | Core. Sim. | Matrix Sim. | Sequence |
| --- | --- | --- | --- | --- | --- | --- | --- |
| Brd4 | \| V$RORA/V$RORA2.01 \|  \| \| --- \| --- \| | 0,82 | 51-73 | + | 0.750 | 0,842 | ggagatttaGCTCagtggtagag |
| Brd4 | V$RORA/ V$RORA2.01 | 0,82 | 56-78 | + | 0.750 | 0,842 | ggagatttaGCTCagtggtagag |
| Brd4 | V$RORA/ V$REV-ERBA.02 | 0,79 | 11-33 | - | 1.000 | 0,825 | caagaactgtGTCAgagagctgg |
| Cdk2ap1 | V$RORA/ V$REV-ERBA.02 | 0,79 | 296-318 | + | 1.000 | 0,791 | cgaaataaatGTCAagaatgggt |
| Sept1 | V$RORA/RORA.01 | 0,85 | 454-476 | - | 1.000 | 0,876 | tgaggttaagGTCAgtgtgggag |
| Sept1 | V$RORA/RORA1.01 | 0,93 | 454-476 | - | 1.000 | 0,941 | tgaggttaaGGTCagtgtgggag |
| Sept1 | V$RORA/REV-ERBA.02 | 0,79 | 87-109 | + | 0.750 | 0,804 | ttctatgtagCTCAgggacctca |
| Sept1 | V$RORA/RORA2.01 | 0,82 | 87-109 | + | 0.750 | 0,849 | ttctatgtaGCTCagggacctca |
| Sept1 | V$RORA/RORGAMMA.01 | 0,80 | 87-109 | + | 0.750 | 0,834 | ttctatgtaGCTCagggacctca |
| Sept1 | V$RORA/REV-ERBA.02 | 0,79 | 270-292 | + | 0.750 | 0,804 | cactatgtagGCCAtggtggcct |
| Sept1 | V$RORA/RORA2.01 | 0,82 | 270-292 | + | 0.750 | 0,846 | cactatgtaGGCCatggtggcct |
| Sept1 | V$RORA/RORGAMMA.01 | 0,80 | 270-292 | + | 0.750 | 0,836 | cactatgtaGGCCatggtggcct |
| Sept1 | V$RORA/REV-ERBA.01 | 0,88 | 920-942 | - | 1.000 | 0,933 | ccccaaaaagGTCAccccaagat |
| Sept1 | V$RORA/RORA.01 | 0,85 | 920-942 | - | 1.000 | 0,851 | ccccaaaaagGTCAccccaagat |
| Sept1 | V$RORA/RORA1.01 | 0,93 | 920-942 | - | 1.000 | 0,949 | ccccaaaaaGGTCaccccaagat |
| Sept1 | V$RORA/RORA2.01 | 0,82 | 920-942 | - | 1.000 | 0,832 | ccccaaaaaGGTCaccccaagat |
| Sept1 | V$RORA/RORGAMMA.01 | 0,80 | 920-942 | - | 1.000 | 0,831 | ccccaaaaaGGTCaccccaagat |
| Sept1 | V$RORA/REV-ERBA.03 | 0,72 | 1139-1161 | - | 0.750 | 0,745 | gaggacgtggGTCCgtgggaaag |
| Snx9 | V$RORA/ V$RORA2.01 | 0,82 | 247-269 | + | 1.000 | 0,829 | ggattcataGGTCaagccttaat |
| Snx9 | V$RORA/ V$REV-ERBA.01 | 0,88 | 14-36 | + | 1.000 | 0,962 | gcagaattggGTCAtctcaggtg |
| Snx9 | V$RORA/ V$REV-ERBA.02 | 0,79 | 554-576 | + | 1.000 | 0,848 | cacaaggtaaGTCActgtgctct |
| Ttc7 | V$RORA/V$REV-ERBA.01 | 0,88 | 630-652 | - | 1.000 | 0,880 | acccactgggGTCAgttgtgcct |
| Ttc7 | V$RORA/V$REV-ERBA.03 | 0,72 | 86-108 | - | 0.750 | 0,736 | caggcagtgaGTCCtagggccag |
| Dnmt1 | V$RORA/V$RORA1.01 | 0,93 | 51-73 | - | 1.000 | 0,944 | tcagtttgaGGTCagccaagtct |
| Dnmt1 | V$RORA/V$RORA1.01 | 0,93 | 154-176 | - | 1.000 | 0,944 | tgagtttgaGGTCagcctgggct |
| Tet2 | V$RORA/ V$RORA1.01 | 0,93 | 44-66 | + | 1.000 | 0,958 | taattttgaGGTCaaaagcagtg |
| Tet2 | V$RORA/ V$RORA1.01 | 0,93 | 225-247 | + | 1.000 | 0,931 | ctagaggaaGGTCaaaccactgt |
| Tet2 | V$RORA/ V$VERBA.01 | 0,92 | 277-299 | + | 1.000 | 0,948 | attggctgaGGTCatgcgtgtgc |
| Tet2 | V$RORA/ V$REV-ERBA.02 | 0,79 | 135-157 | - | 1.000 | 0,826 | gagaaaatgtGTCAggagaacct |
| Tet2 | V$RORA/ V$REV-ERBA.01 | 0,88 | 476-498 | - | 1.000 | 0,897 | gttaaaaccgGTCAtcagaagtg |
|  |  |  |  |  |  |  |  |
| Gene symbol | Family/Matrix | Opt. | Position | Strand | Core. Sim. | Matrix Sim. | Sequence |
| Dnmt3a | V$RORA/ V$RORA1.01 | 0,93 | 432-454 | - | 1.000 | 0,949 | agaactccaGGTCacgtgggccc |
| Dnmt3a | V$RORA/ V$REV-ERBA.03 | 0,72 | 122-144 | - | 0.750 | 0,729 | cccatagtctTTCAcctggccag |
| Dnmt3a | V$RORA/ V$REV-ERBA.03 | 0,72 | 409-431 | + | 0.750 | 0,750 | tgcaactcagGACActggggcac |
| Dnmt3a | V$RORA/ V$RORA2.01 | 0,82 | 62-84 | + | 0.750 | 0,849 | taggaactaGCTCactgcaggtg |
| Dnmt3a | V$RORA/ V$RORA2.01 | 0,82 | 154-176 | + | 0.750 | 0,833 | tgtcatttaGTTCaaccttccac |
| Dnmt3a | V$RORA/ V$RORA2.01 | 0,82 | 18-40 | - | 0.750 | 0,824 | ctccatctaGGGCacatgtcttc |
| Dnmt3a | V$RORA/ V$VERBA.01 | 0,92 | 393-415 | + | 1.000 | 0,936 | caggccttaGGTCacactgtcgg |
| Dnmt3a | V$RORA/ V$VERBA.01 | 0,92 | 470-492 | + | 1.000 | 0,936 | caggccttaGGTCacactgttgg |
| Dnmt3a | V$RORA/ V$REV-ERBA.03 | 0,72 | 517-539 | + | 0.750 | 0,735 | agctcactagCTCAcagtggcat |
| Dnmt3a | V$RORA/ V$REV-ERBA.03 | 0,72 | 286-308 | + | 0.750 | 0,734 | ggggcaggaaGCCActggggcag |
| Dnmt3a | V$RORA/ V$REV-ERBA.03 | 0,72 | 294-316 | + | 0.750 | 0,735 | aagccactggGGCAgtgtggcac |
| Dnmt3a | V$RORA/ V$VERBA.01 | 0,92 | 488-510 | + | 1.000 | 0,936 | gaaacctaaGGTCaaggagatca |
| Dnmt3b | V$RORA/ V$RORA2.01 | 0,82 | 16-38 | + | 0.750 | 0,821 | gccaagctaGGTGaaagcaggaa |
| Dnmt3b | V$RORA/ V$REV-ERBA.03 | 0,72 | 452-474 | - | 0.750 | 0,745 | cctcatcttgATCActaaggcaa |
| Dnmt3b | V$RORA/ V$REV-ERBA.02 | 0,89 | 537-559 | + | 1.000 | 0,829 | cggaatctgaGTCAacggttatt |
| Tet1 | V$RORA/V$REV-ERBA.02 | 0,79 | 295-317 | - | 1.000 | 0,876 | gacaatttgtGTCAcggccatct |
| Tet1 | V$RORA/V$REV-ERBA.03 | 0,72 | 415-437 | + | 1.000 | 0,812 | ggccatccaaGTCAatgggccaa |
| Tet1 | V$RORA/V$RORA2.01 | 0,82 | 509-531 | + | 0.750 | 0,855 | ctgaaaataGGTGatcttttgac |
| Tet1 | V$RORA/V$RORA2.01 | 0,82 | 531-553 | + | 0.750 | 0,866 | caataaataGGACatgaacaaaa |
| Tet1 | V$RORA/V$RORA.01 | 0,85 | 377-399 | - | 1.000 | 0,852 | gatagaagagGTCAtgtgatgac |
| Tet1 | V$RORA/V$REV-ERBA.01 | 0,88 | 810-832 | + | 1.000 | 0,894 | gtgagattagGTCAagattgcct |
| Tet1 | V$RORA/V$RORA2.01 | 0,82 | 192-214 | - | 0.750 | 0,827 | ggtaaagtgGCTCaaatttttat |
| Tet1 | V$RORA/V$REV-ERBA.01 | 0,88 | 387-409 | - | 1.000 | 0,885 | gccaagcttgGTCAcctgagttt |
| Tet1 | V$RORA/V$REV-ERBA.02 | 0,79 | 138-160 | - | 0.750 | 0,803 | ggtaagatggCTCAgtggacgaa |
| Tet1 | V$RORA/V$RORA1.01 | 0,93 | 326-348 | + | 1.000 | 0,931 | cattcacgaGGTCactcaaaact |
| Tet3 | V$RORA/V$RORA1.01 | 0,93 | 238-260 | + | 1.000 | 0,935 | ctacacaaaGGTCactggcccct |
| Tet3 | V$RORA/V$REV-ERBA.01 | 0,88 | 61-83 | - | 1.000 | 0,901 | tccaagccagGTCAgtcctccct |
| Opn4 | V$RORA/V$VERBA.01 | 0,92 | 876-898 | - | 1.000 | 0,966 | agacagtcaGGTCacggctgcca |
| Opn4 | V$RORA/V$RORA2.01 | 0,82 | 1450-1472 | - | 0.750 | 0,852 | ggagaaataGCTCaggggttaag |
|  |  |  |  |  |  |  |  |
