# Supplementary material for: Cell–cell coupling and DNA methylation abnormal phenotypes in the after-hours mice
Source: Epigenetics Chromatin. 2021 Jan 6;14:1. doi: 10.1186/s13072-020-00373-5 (PMC7789812; doi:10.1186/s13072-020-00373-5)
Supplement: Supplementary file 11 — Additional file 11: Table S7. Math inspector table of ROR family binding sites. [file 13072_2020_373_MOESM11_ESM.docx]

| Tag name | Sequence (5`-3`) |
| --- | --- |
| PE1.0 | AATGATACGGCGACCACCGAGATCTACACTCTTTCCCTACACGACGCTCTTCCGATC*T |
| PE2.1 | CAAGCAGAAGACGGCATACGAGA*T |
| RBBS_S tag 1 | CAAGCAGAAGACGGCATACGAGATNNNNNTGATGAGATCGGTCTCGGCATTCCTGCTGAACCGCTCTTCCGAT*C |
| RBBS_S tag 2 | CAAGCAGAAGACGGCATACGAGATNNNNNATCGGAGATCGGTCTCGGCATTCCTGCTGAACCGCTCTTCCGAT*C |
| RBBS_S tag 3 | CAAGCAGAAGACGGCATACGAGATNNNNNCTAAGAGATCGGTCTCGGCATTCCTGCTGAACCGCTCTTCCGAT*C |
| RBBS_S tag 4 | CAAGCAGAAGACGGCATACGAGATNNNNNGTCAGAGATCGGTCTCGGCATTCCTGCTGAACCGCTCTTCCGAT*C |
| RBBS_S tag 5 | CAAGCAGAAGACGGCATACGAGATNNNNNCTGTGAGATCGGTCTCGGCATTCCTGCTGAACCGCTCTTCCGAT*C |
| RBBS_S tag 6 | CAAGCAGAAGACGGCATACGAGATNNNNNTGGCGAGATCGGTCTCGGCATTCCTGCTGAACCGCTCTTCCGAT*C |
| RBBS_S tag 7 | CAAGCAGAAGACGGCATACGAGATNNNNNTCTGGAGATCGGTCTCGGCATTCCTGCTGAACCGCTCTTCCGAT*C |
| RBBS_S tag 8 | CAAGCAGAAGACGGCATACGAGATNNNNNAAGTGAGATCGGTCTCGGCATTCCTGCTGAACCGCTCTTCCGAT*C |
| RBBS_S tag 9 | CAAGCAGAAGACGGCATACGAGATNNNNNGATCGAGATCGGTCTCGGCATTCCTGCTGAACCGCTCTTCCGAT*C |
| RBBS_S tag 10 | CAAGCAGAAGACGGCATACGAGATNNNNNGCTAGAGATCGGTCTCGGCATTCCTGCTGAACCGCTCTTCCGAT*C |
| RBBS_S tag 11 | CAAGCAGAAGACGGCATACGAGATNNNNNAGCCGAGATCGGTCTCGGCATTCCTGCTGAACCGCTCTTCCGAT*C |
| RBBS_S tag 12 | CAAGCAGAAGACGGCATACGAGATNNNNNCAAGGAGATCGGTCTCGGCATTCCTGCTGAACCGCTCTTCCGAT*C |
| IlAdap Methyl PE1 | AcAcTcTTTcccTAcAcGAcGcTcTTccGATc*T |
| IlAdap Methyl PE2 | pGATcGGAAGAGcGGTTcAGcAGGAATGccGA*G |
| *= Phosphorotiate bond, N = random nucleotides, c= methylated cytosines, p= 5` phosphate | |

**Additional file 11: Table S7: RRBS primers**
