# Supplementary material for: Cell–cell coupling and DNA methylation abnormal phenotypes in the after-hours mice
Source: Epigenetics Chromatin. 2021 Jan 6;14:1. doi: 10.1186/s13072-020-00373-5 (PMC7789812; doi:10.1186/s13072-020-00373-5)
Supplement: Supplementary file 12 — Additional file 12: Table S6. RT-qPCR primer sequences. [file 13072_2020_373_MOESM12_ESM.docx]

**Additional file 12: Table S6: RT-qPCR primers**

| Primer Set | Sense primer (5`-3`) | Antisense primer (3`-5`) |
| --- | --- | --- |
| Actin | AAGTGGTTACAGGAAGTCC | ATAATTTACACAGAAGCAATGC |
| GAPDH | GAACATCATCCCTGCATCCA | CCAGTGAGCTTCCCGTTCA |
| HPRT1 | TGAGGCGGCGAGGGAGAG | AAGCGGTCTGAGGAGGAAGC |
| Per1 | CCCCTGCCTCCCAGTGA | CTGAAAGTGCATCCTGATTGGA |
| Per2 | AGCTACACCACCCCTTACAAGCT | GACACGGCAGAAAAAAGATTTCTC |
| Cry1 | GCTATGCTCCTGGAGAGAACGT | TGTCCCCGTGAGCATAGTGTAA |
| Cry2 | TGACCTACAGAGAATCATCGAACTG | GGCTGATGAGGGCCTGAA |
| Rev-ERBɑ | CGTTCGCATCAATCGCAACC | GATGTGGAGTAGGTGAGGTC |
| Dnmt1 | GGAAGGCTACCTGGCTAAAGTCAAG | ACTGAAAGGGTGTCACTGTCCGAC |
| Dnmt2 | AATGATTCTTGATGATTGATTCTT | CTGTGTTGCTGTCTACTG |
| Dnmt3a | TGGAGAATGGCTGCTGTGTGAC | CACTCATCCCGTTTCCGTTTG |
| Dnmt3b | AGTGACCAGTCCTCAGACACGAAG | ATCAGAGCCATTCCCATCATCTAC |
| Tet1 | CCATTCTCACAAGGACATTCACA | GCAGGACGTGGAGTTGTTCA |
| Tet2 | GCCATTCTCAGGAGTCACTGC | ACTTCTCGATTGTCTTCTCTATTGAGG |
| Tet3 | GGTCACAGCCTGCATGGACT | AGCGATTGTCTTCCTTGGTCAG |
| Brd4 | CAAGTCATCCAGCATCAC | TGTATCATAAGCGGAGAGG |
| Cdk2ap1 | GCTGCTGAGTGACTATGGG | TCTGAGGCACCTGGCTAT |
| Opn4 | CATTATCTACGCCATCACTCA | GCCTGATACACCGAGAAG |
| Sept1 | ATCGCAAGTCTGTCAAGAAG | CGTAGAGGTTGGTGAGGAA |
| Snx9 | CCGAATGTTGGTGGAGGA | TGTTGGCAGAAGATGAGTTG |
| Ttc7 | CACATCCGCCTGACTGAG | ATACCTGAGCCACCCAAGA |
| Adam11 | CCCAGGTGAGTTTCGTCAT | AGGACAGGAGGTGATGGT |
| Six6 | GTGGGCAACTGGTTCAAAAA | AGATGTCGCACTCACTGTCG |
| All the primers` sequences are presented with a 5`-3` orientation | | |
