# Supplementary material for: Cell–cell coupling and DNA methylation abnormal phenotypes in the after-hours mice
Source: Epigenetics Chromatin. 2021 Jan 6;14:1. doi: 10.1186/s13072-020-00373-5 (PMC7789812; doi:10.1186/s13072-020-00373-5)
Supplement: Supplementary file 13 — Additional file 13: Table S2. RRBS primer sequences. [file 13072_2020_373_MOESM13_ESM.docx]

| Genotype | Total  sequences | Unique  alignments | Mapping  efficiency | CpG  methylation % | CHG  methylation % | CHH  methylation % |
| --- | --- | --- | --- | --- | --- | --- |
| Wt 1 | 14,255,858 | 8,817,506 | 61.9 | 23.4 | 1.4 | 1.6 |
| Wt 2 | 18,705,544 | 11,218,745 | 60.0 | 23.4 | 1.4 | 1.5 |
| Wt 3 | 18,409,493 | 11,411,534 | 62.0 | 23.0 | 1.4 | 1.5 |
| Wt 4 | 13,434,827 | 7,858,914 | 58.5 | 19.2 | 1.5 | 1.5 |
| Wt 5 | 14,932,240 | 9,076,340 | 60.8 | 22.6 | 1.7 | 1.8 |
| Wt 6 | 15,300,699 | 9,492,079 | 62.0 | 22.4 | 1.6 | 1.7 |
| Homo 1 | 17,472,882 | 11,178,992 | 64.0 | 22.7 | 1.2 | 1.4 |
| Homo 2 | 18,327,326 | 11,747,505 | 64.1 | 22.5 | 1.1 | 1.2 |
| Homo 3 | 18,083,133 | 11,564,362 | 64.0 | 22.3 | 1.2 | 1.4 |
| Homo 4 | 48,272,892 | 31,677,602 | 65.6 | 26.0 | 1.1 | 1.3 |
| Homo 5 | 31,868,240 | 20,360,818 | 63.9 | 24.5 | 1.0 | 1.3 |
|  |  |  |  |  |  |  |

**Additional file 13: Table S2: RRBS libraries statisitic**
